# A New Insight into the Molecular Mechanism of the Reaction between 2-Methoxyfuran and Ethyl (*Z*)-3-phenyl-2-nitroprop-2-enoate: An Molecular Electron Density Theory (MEDT) Computational Study

**DOI:** 10.3390/molecules29204876

**Published:** 2024-10-14

**Authors:** Mikołaj Sadowski, Ewa Dresler, Aneta Wróblewska, Radomir Jasiński

**Affiliations:** 1 Department of Organic Chemistry and Technology, Cracow University of Technology, Warszawska 24, 31-155 Kraków, Poland; mikolaj.sadowski@doktorant.pk.edu.pl; 2 Łukasiewicz Research Network—Institute of Heavy Organic Synthesis “Blachownia”, Energetyków 9, 47-225 Kedzierzyn-Kozle, Poland; ewa.dresler@icso.lukasiewicz.gov.pl; 3 Department of Organic Chemistry, University of Lodz, Tamka 12, 91-403 Lodz, Poland; aneta.wroblewska@chemia.uni.lodz.pl

**Keywords:** nitroalkenes, Diels–Alder, hetero Diels–Alder, cycloaddition, furan, MEDT

## Abstract

The molecular mechanism of the reaction between 2-methoxyfuran and ethyl (*Z*)-3-phenyl-2-nitroprop-2-enoate was investigated using wb97xd/6-311+G(d,p)(PCM) quantum chemical calculations. It was found that the most probable reaction mechanism is fundamentally different from what was previously postulated. In particular, six possible zwitterionic intermediates were detected on the reaction pathway. Their formation is determined by the nature of local nucleophile/electrophile interactions. Additionally, the channel involving the formation of the *exo*-nitro Diels–Alder cycloadduct was completely ruled out. Finally, the electronic nature of the five- and six-membered nitronates as potential TACs was evaluated.

## 1. Introduction

The most universal protocol for the preparation of six-membered carbocyclic molecules is the [4+2] cycloaddition (42CA) process involving conjugated dienes [1,2,3,4,5] discovered by *Otto Diels* and *Kurt Alder* [6]. It is important to note that analogous processes exist in organic chemistry, involving heteroanalogs of dienes, such as conjugated nitroalkenes [7,8,9,10], nitrosocompounds [11,12,13], azoalkenes [14], and others [15,16,17,18]. Among a wide range of 42CA-type transformations, reactions involving cyclopentadiene, furan, and thiophene play a particularly important role. These reactions provide an efficient protocol for the synthesis of norbornene derivatives, as well as their heterocyclic analogs, which are of significant practical interest [19,20,21,22,23].

**Scheme 1:**
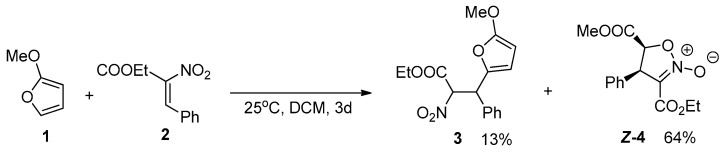
Experimental results of the reaction between 2-methoxyfuran (**1**) and ethyl (*Z*)-3-phenyl-2-nitroprop-2-enoate (**2**).

Although the heteroaromatic furan molecule is not formally a conjugated diene, it exhibits reactivity similar to cyclopentadiene according to the 42CA scheme [24,25,26]. This type of transformation is particularly accelerated by the significant difference in the global electrophilicities of the cycloaddition components [27,28]. Some time ago, Itoh and *Kishimoto* [29] reported the results of experimental research on the reaction between 2-methoxyfuran (**1**) and ethyl (*Z*)-3-phenyl-2-nitroprop-2-enoate (**2**). Specifically, in the post-reaction mixture, the authors detected two major products: 2-metxohy-5-(2-carboethoxy-2-nitro-1-phenylethyl)-furane (**3**) and *4,5-cis*-3-carboethoxy-4-phenyl-5-carbomethoxy-isoxazoline 2-oxide (***Z*-4**) (Scheme 1).

**Scheme 2:**
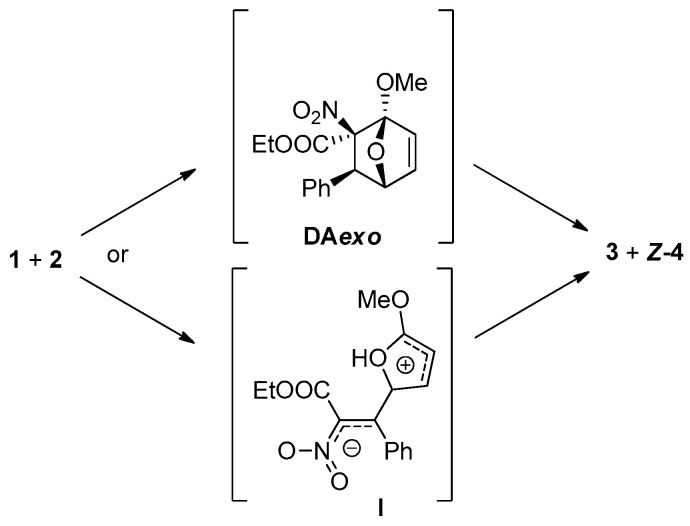
Postulated mechanisms for the reaction between 2-methoxy (**1**) and ethyl (*Z*)-3-phenyl-2-nitroprop-2-enoate (**2**).

Based on these observations, the authors proposed two alternative mechanisms for the formation of the detected products (Scheme 2). The first approach involves the formation of a Diels–Alder type *exo*-nitro adduct (**DA*exo***) in the initial reaction stage. In this scheme, the **DA*exo*** molecular system is treated as a common intermediate for the formation of the target products **3** and ***Z*-4**. The alternative approach suggests that a (Z)witterionic adduct is formed in the first reaction stage. This intermediate can then be converted to the detected products via respective rearrangement pathways.

Unfortunately, the mechanistic considerations presented should be treated only as intuitive propositions rather than definitive explanations. This proposal contains several weak points, as follows, and many key issues were not thoroughly analyzed, necessitating reexamination and deeper investigation:

(i) The formation of the detected adducts via an intermediate stage is rather evident. The number of possible intermediates is, however, substantially higher. Next, the detected products may form via a common intermediate or through two different types of intermediates. Such scenarios have recently been analyzed in reactions between conjugated dienes and alkenes [30].
(ii) The authors assumed a priori the formation of *exo*-type Diels–Alder cycloadducts. However, many experimental results indicate that in Diels–Alder reactions between conjugated dienes and conjugated nitroalkenes, the *endo*-nitro isomer is always preferred [31,32]. Unfortunately, the possibility of forming these types of cycloadducts was not considered in the mechanistic discussion.
(iii) Assuming a zwitterionic mechanism for the title reaction, not just one, but six isomeric zwitterionic intermediates should be considered [33]. Different zwitterions may convert to the same or different final products. Furthermore, the mutual conversion of zwitterions through a rotation of the single bond within >C-C-NO_2_ moiety is feasible and should be considered.
(iv) In reactions involving conjugated nitroalkenes, the classical “carbo” Diels–Alder scheme can compete with the hetero Diels–Alder reaction, where the nitroalkene acts as a heteroanalog of the diene [9,34].
(v) Assuming the formation of the Diels–Alder product within the initial reaction stage, a one-step cycloaddition mechanism cannot be assumed a priori. Recently, many examples of stepwise Diels–Alder reactions have been shown to proceed through the formation of biradical or zwitterionic intermediates [35].
(vi) Although the stereoconfiguration of *4,5-cis*-3-carboethoxy-4-phenyl-5-carbomethoxy-isoxazoline 2-oxide was fully established on the basis of the RTG experiment, the stereoconfiguration of the Michael-type adduct (**3**) remains unclear. In practice, more than one structure of this type of adduct is possible [32,36].

The authors identified only 77% of the post-reaction mixture, and the composition of the residue is unknown. This fraction can include product(s) other than those presented in Scheme 1.

**Scheme 3:**
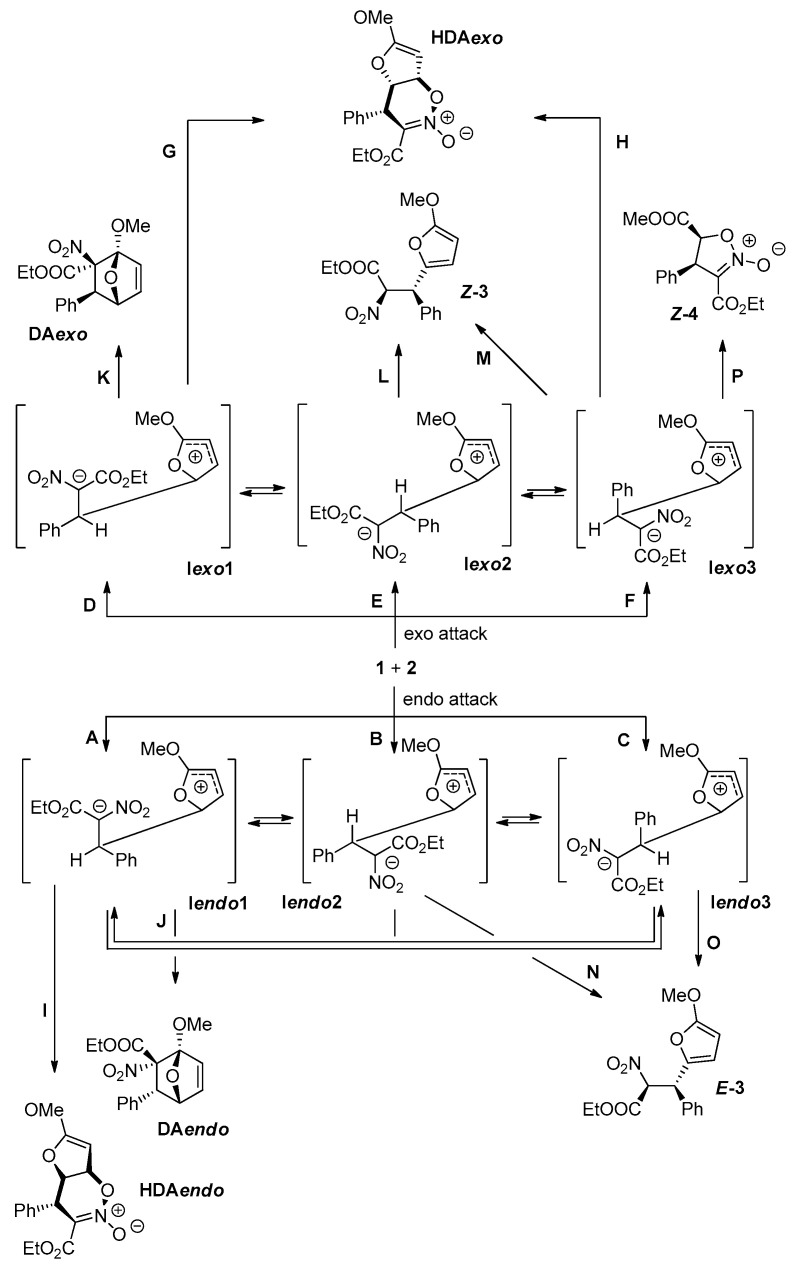
Considered paths of the reaction between 2-methoxyfuran (**1**) and ethyl (*Z*)-3-phenyl-2-nitroprop-2-enoate (**2**).

Therefore, in this study, we aimed to address and explain all of the mechanistic aspects mentioned above. Specifically, we explored all theoretically possible pathways of the reaction system’s transformation (Scheme 3). For this purpose, we applied Density Functional Theory (DFT) calculations at the wb97xd/6-311+G(d,p) level of theory.

## 2. Results and Discussion

### 2.1. Electronic Interactions

According to the current understanding [37,38], changes in electron density along a chemical reaction are responsible for the chemical reactivity of organic molecules. In practice, for bimolecular organic polar processes, the formation of new bonds is determined by interactions between the most electrophilically activated reaction center of the first molecule and the most nucleophilically activated center of the second [39,40]. This approach has been successfully applied to predict the reactivity of many polar components as well as the regioselectivity of various organic reactions [41,42,43,44,45,46].

The relevant descriptors for the components of the reaction in question are summarized in Table 1. It was found that the electronic chemical potential (*Z*)-3-phenyl-2-nitroprop-2-enoate (**2**) is −4.51 eV. As a result, the reaction with 2-methoxyfuran (**1**) should be determined by the electron density transfer from the furan molecule to the nitroalkene. Thus, according to Domingo’s terminology [47], this reaction should be classified as a Forward Electron Density Flux (FEDF) process. Moreover, the global electrophilicity of the nitroalkene (2.24 eV) is substantially higher than the analogous parameter estimated for methoxyfuran (0.39 eV). Therefore, the key interatomic interactions must be regarded as polar in nature.

**Scheme 4:**
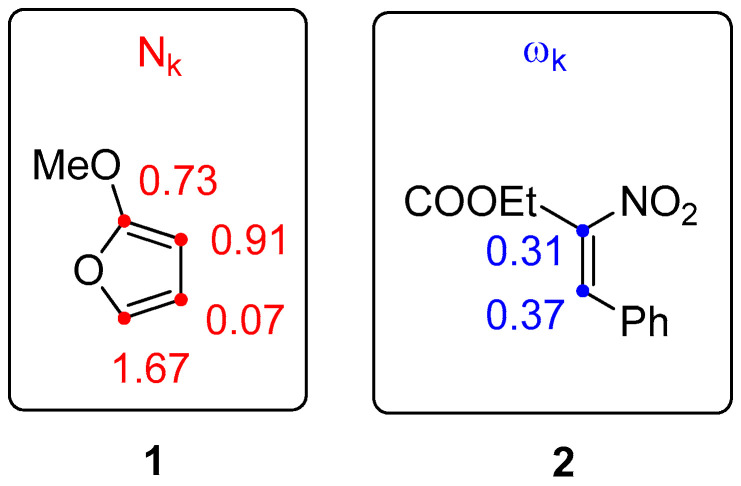
Local electronic properties of 2-methoxyfuran (**1**) and ethyl (*Z*)-3-phenyl-2-nitroprop-2-enoate (**2**).

As a consequence, the regio-orientation in the first stage of the reaction is primarily determined by the interaction between the C5 nucleophilic atom in the furan **1** molecule and the beta-carbon atom in the nitrovinyl moiety of (**2**) (Scheme 4). This type of mutual orientation aligns perfectly with the further exploration of the reaction profiles and is consistent with the observed regioselectivity of the reaction [29].

### 2.2. Energetic Considerations

For all transformations leading to the target products (Diels–Alder adducts, hetero Diels–Alder products, Michael adducts, and five-membered internal nitronates), the initial reaction stage always involves the formation of the respective pre-reaction molecular complex MC (Scheme 3, Table 2). Depending on the mutual orientation of the reactant molecules, six structures of pre-reaction complexes are possible: **MCA**, **MCB**, and **MCC** within the *endo* approach, and **MCD**, **MCE**, and **MCF** within the *exo* approach, respectively. The formation of these MCs is associated with a reduction in the enthalpy of the reaction system by several kcal/mol. However, at the same time, the entropy of the reaction system is significantly reduced. Consequently, the Gibbs free energies for the formation of the respective MCs are positive, which excludes the possibility of MCs existing as relatively stable intermediates.

Further conversion of the pre-reaction complexes is possible via six different transition states (**TSA**, **TSB**, and **TSC** within the *endo* approach, and **TSD**, **TSE**, and **TSF** within the *exo* approach, cartesian coordinates for transition states can be found in the supplementary materials), leading to the respective acyclic adducts. These processes are associated with an increase in the enthalpy of the reaction system by 14.4–17.3 kcal/mol relative to the corresponding MC. The nature of the localized saddle points was confirmed by vibrational analysis and intrinsic reaction coordinate (IRC) computations (see Computational Details section). In all six reaction channels, the IRC trajectories connect the transition states (TSs) to the valleys of the respective acyclic adducts, which can be considered reaction intermediates (**I*endo*1**, **I*endo*2**, and **I*endo*3** for paths **A**, **B**, and **C**, respectively; and **I*exo*1**, **I*exo*2**, and **I*exo*3** for paths **D**, **E**, and **F**, respectively). It is important to note that all attempts to locate reaction channels leading directly to the DA or HDA type of adducts were unsuccessful.

All localized intermediates are labile, allowing free rotation around the C4-C5 single bond. These processes require only low activation energy (Table 2). It is interesting to note that, in the case of **I*exo*1**, gradual rotation in the direction that theoretically should lead to the formation of **I*exo*3** actually results in the formation of the hetero Diels–Alder adduct **HDA*exo*** (path **G** in Scheme 3). This process occurs via the transition state **TSG** and requires an activation energy of approximately 1.4 kcal/mol. Similarly, the gradual rotation of **Iexo3**, following the path that might lead to **I*exo*1**, also results in the formation of the same **HDA*exo*** (path **H** in Scheme 3). Both processes proceed via the common transition state **TSG**.

Alternatively, the other hetero Diels–Alder adduct can be formed based on the *endo*-isomeric intermediate. Specifically, **I*endo*1** can cyclize to form **HDA*endo***. This process requires a Gibbs free energy of activation of about 2 kcal/mol and proceeds via the **TSI** transition state.

A competitive channel for the conversion of intermediates **I*exo*1** and **I*exo*1** involves cyclization reactions leading to the formation of respective nitronorbornene molecular systems via pathways **J** and **K** (yielding products **DA*endo*** and **DA*exo***, respectively). From a kinetic point of view, the formation of the carbocyclic norbornene skeletons is relatively more challenging than cyclization to the hetero Diels–Alder products. Furthermore, thermodynamic factors exclude the possibility of these norbornenes being stable products. Thus, it is unlikely that nitronorbornenes are present in the unidentified part of the post-reaction mixture. It should also be emphasized that both kinetic and thermodynamic factors favor the formation of **DA*endo*** over **DA*exo***.

The next possible transformation of the intermediates involves the formation of Michael-type adducts (paths **L**, **M**, **N**, and **O**). In the reaction system considered, two isomeric forms of this skeleton are possible: ***Z*-3** and ***E*-3** (Scheme 3). As previously mentioned, the isomerism of the obtained Michael adduct has not been definitely assigned. From a kinetic perspective, both transformations require relatively high Giggs free energies of activation. However, it should be emphasized that *Itoh* and *Kishimoto* [29] conducted the synthesis under thermodynamic control. We found that, from a thermodynamic standpoint, adducts ***Z*-3** and ***E*-3** are more stable than the Diels–Alder and/or hetero Diels–Alder adducts. This observation correlates well with the experimental results, as these products were identified in the post-reaction mixture rather than the Diels–Alder and/or hetero Diels–Alder adducts.

The final reaction pathway considered is the formation of the nitronate ***Z*-4**. This is possible only through the rearrangement of the intermediate **I*exo*3** and actually requires a Gibbs free energy of activation of about 9 kcal/mol. Notably, the ***Z*-4** product is the most thermodynamically stable of all of the theoretically possible reaction products. Consequently, under thermodynamic control, ***Z*-4** should be identified as the major product in the post-reaction mixture. This conclusion aligns perfectly with the experimental results reported by *Itoh* and *Kishimoto* [29].

### 2.3. Critical Structures

**Figure 1:**
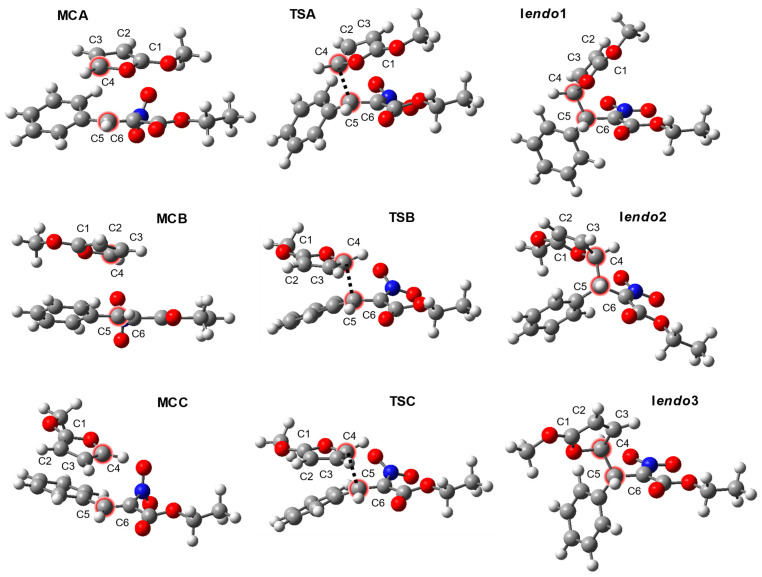
Views of key structures for the formation of zwitterionic intermediates via the *endo*-attack of 2-methoxyfuran (**1**) on the ethyl (*Z*)-3-phenyl-2-nitroprop-2-enoate (**2**) molecule according to wb97xd/6-311+G(d,p) (PCM) calculations.

First, we analyzed the nature of the pre-reaction molecular complexes **MCs** (Figure 1). In these types of complexes, the substructures of the reactants adopt orientations that determine their subsequent conversion to the respective intermediate **I**. Thus, these localized structures should be considered as orientation complexes. It should be noted that within the framework of MCs, no new sigma bonds are formed. The distances between the substructures exceed the typical range for sigma bonds in transition states [7,43,48,49,50]. Additionally, the geometries of the substructures are nearly identical to those of the individual reactants. At this stage, the substructures are stabilized by electrostatic interactions, but no significant electron density transfer occurs between them (GEDT = 0.00e). Similar types of pre-reaction molecular complexes have been recently observed in other bimolecular organic reactions [41,51,52,53,54].

**Figure 2:**
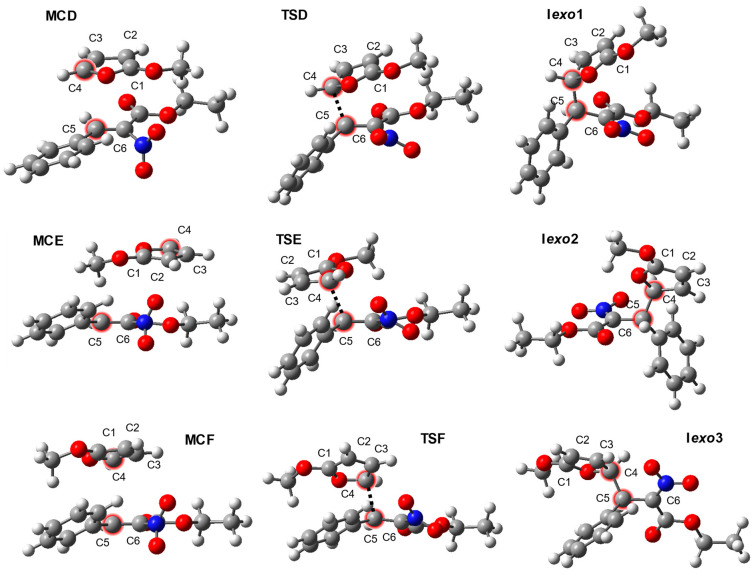
Views of key structures for the formation of zwitterionic intermediates via the *exo*-attack of 2-methoxyfuran (**1**) on the ethyl (*Z*)-3-phenyl-2-nitroprop-2-enoate (**2**) molecule according to wb97xd/6-311+G(d,p) (PCM) calculations.

As the reaction coordinate progresses, the gradual reduction in the distances between the substructures leads to the formation of the respective transition states (**TSA**, **TSB**, and **TSC** for the *endo* approach, and **TSD**, **TSE**, and **TSF** for the *exo* approach). In particular, the C5-C6 distance (Figure 2) decreases most rapidly, reaching approximately 2–2.1 Å. This observation aligns with the local nucleophile/electrophile interactions discussed earlier. In all TSs, electron density transfer between substructures is observed (GEDT = 0.20e, 0.33e, 0.35e, 0.20e, 0.48, and 0.48e for **TSA**, **TSB**, **TSC, TSD**, **TSE**, and **TSF**, respectively). This confirms, without any doubts, the polar nature of the process.

The direct products of the transformation of the mentioned TSs are respective acyclic intermediates (**I*endo*1**, **I*endo*2**, and **I*endo*3** for paths **A**, **B**, and **C**; and **I*exo*1**, **I*exo*2**, and **I*exo*3** for paths **D**, **E**, and **F**). Their nature was explored on the basis of an ELF study of the model molecule **I*endo*1**.

**Figure 3:**
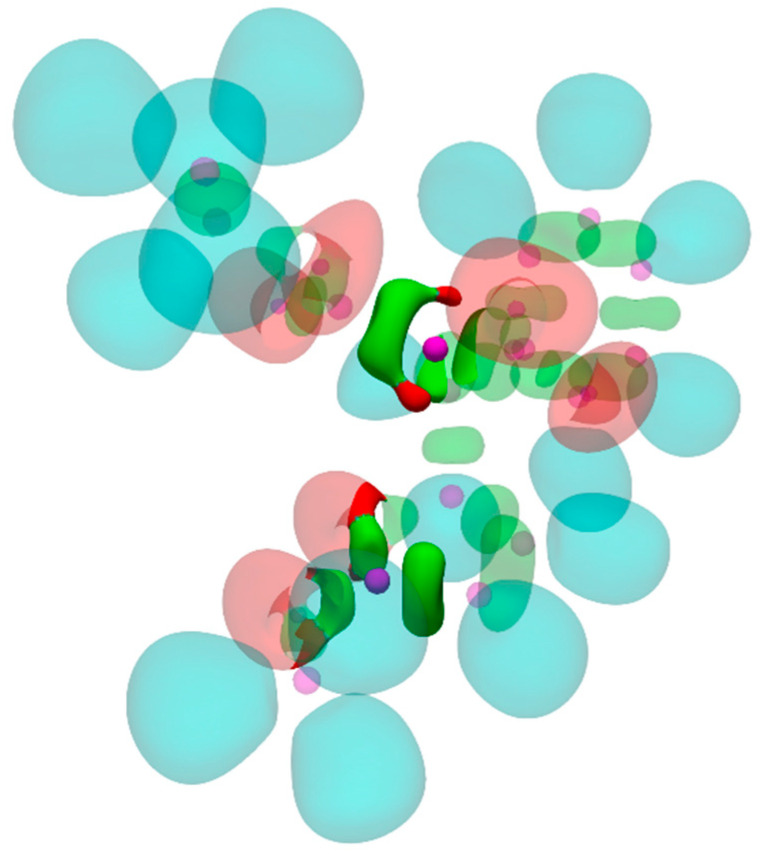
Topology of **I*endo*1** ELF, rendered at an isovalue of 0.8. Core basins are shown in magenta, protonated basins in cyan, disynaptic basins in green, and monosynaptic basins in red. Parts of the function significant for intermediate-type identification are depicted as solid, while the rest are translucent.

**Figure 4:**
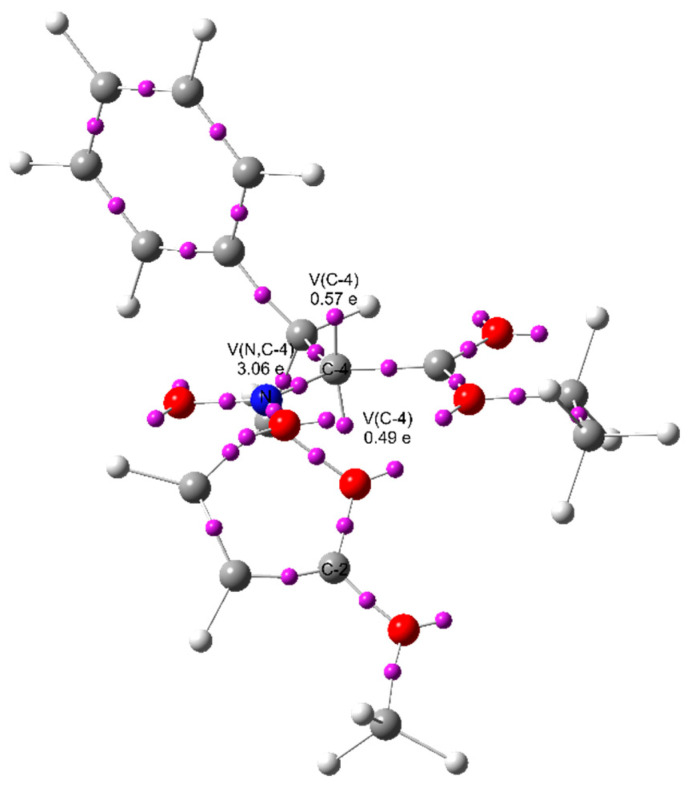
Positions and populations of significant ELF attractors of unprotonated basins in **I*endo*1**.

**Scheme 5:**
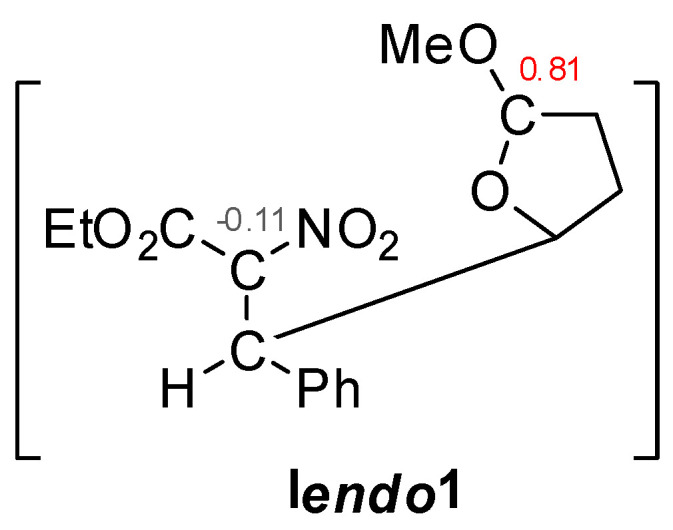
Natural charges of significant atoms in the structure **I*endo*1**, calculated using the wb97xd/6-311+G(d,p) (PCM) level of theory.

In particular, the ELF topological analysis of **I*endo*1** revealed two irreducible [55] monosynaptic valence basins (Figure 3) at the C-4 carbon atom (Figure 4), one with a population of 0.57e and the other with 0.49e (Figure 4). The C-4 carbon atom also has a charge of −0.11 (Scheme 5), while the other reaction center, C-2, is strongly positively charged (0.84e) (Scheme 5). Although the valence basins and the slightly negative charge at C-4 might suggest a carbene-type intermediate [56], the total population of both V(C-4), approximately 1e, is insufficient to confirm a carbene structure. Combined with the highly positive charge at C-2, this suggests that the intermediate is a zwitterion. The influence of a strongly electron-withdrawing (EW) nitro-group on the C-4 atom might explain the slightly negative charge at the C-4 atom. The V(N,C-4) population is 3.06e, which indicates a strongly overpopulated single bond, reinforcing the influence of the EW nitro-group on the C-4 atom charge.

**Figure 5:**
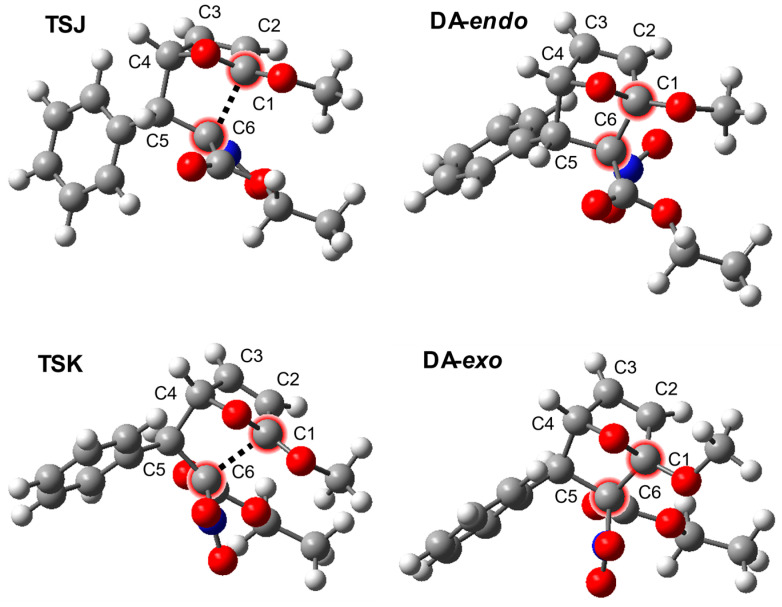
Views of key structures for the formation of DA adducts from isomeric zwitterions formed in the reaction between 2-methoxyfuran (**1**) and ethyl (*Z*)-3-phenyl-2-nitroprop-2-enoate (**2**) according to wb97xd/6-311+G(d,p) (PCM) calculations.

**Figure 6:**
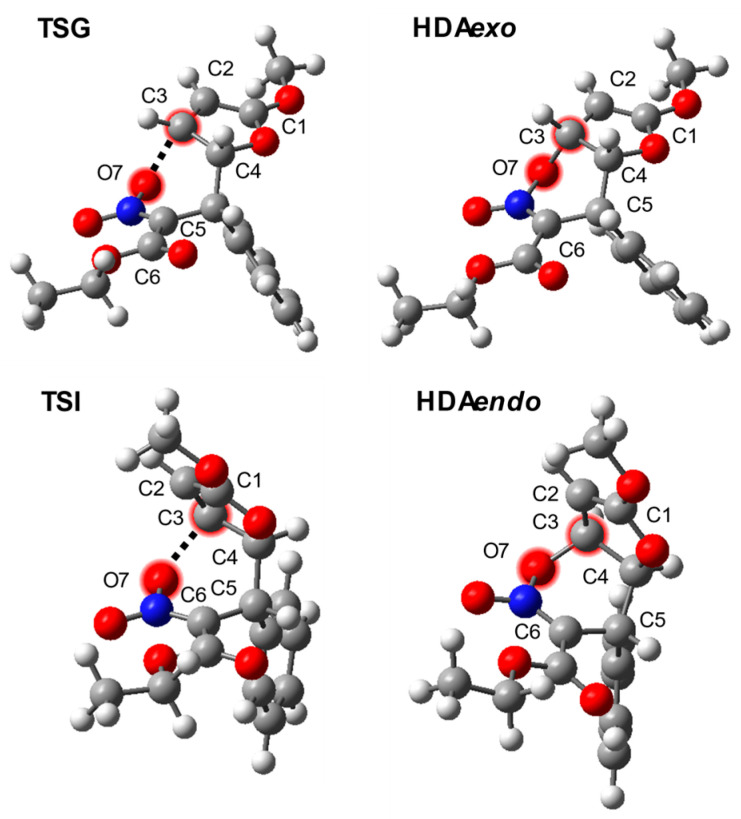
Views of key structures for the formation of HDA adducts from isomeric zwitterions formed in the reaction between 2-methoxyfuran (**1**) and ethyl (*Z*)-3-phenyl-2-nitroprop-2-enoate (**2**) according to wb97xd/6-311+G(d,p) (PCM) calculations.

A separate group of detected transition states are structures associated with cyclization processes. Two types of these TSs are possible in the context of this reaction. The first group involves TSs leading to the formation of nitronorbornene skeletons (**TSJ** and **TSK** for the *endo* and *exo* approaches, respectively). In these TSs, a new C1-C6 single bond forms (C1–C6—Figure 5). The second group involves TSs leading to the formation of 1,2-oxazine N-oxide structures (**TSJ** and **TSK** for *endo* and *exo* approaches, respectively), where a new C3-O7 single bond is formed (Figure 6).

**Figure 7:**
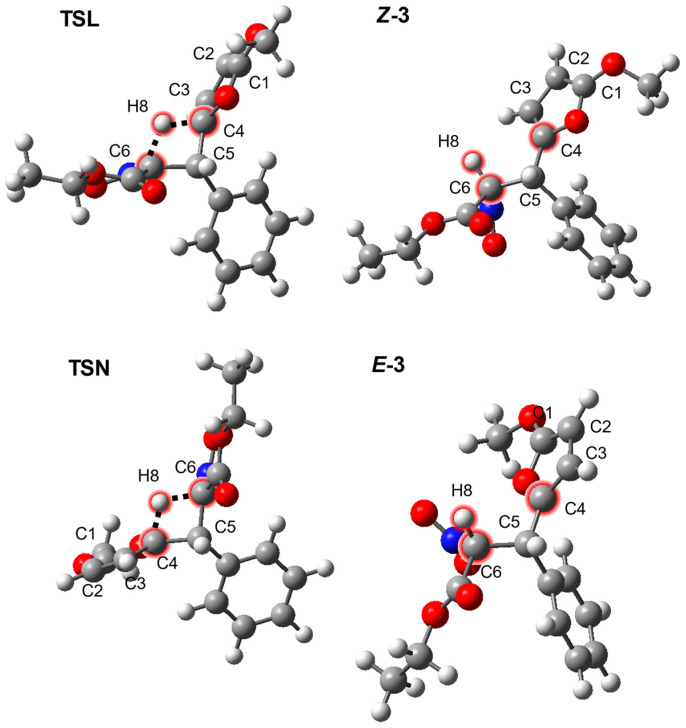
Views of key structures for the formation of Michael-type adducts from isomeric zwitterions formed in the reaction between 2-methoxyfuran (**1**) and ethyl (*Z*)-3-phenyl-2-nitroprop-2-enoate (**2**) according to wb97xd/6-311+G(d,p) (PCM) calculations.

For comparison, the transition states leading from respective intermediates to Michael-type products (**TSL** and **TSN**, Figure 7) exhibit characteristics of transition states typical of [1.3]-sigmatropic hydrogen shifts [57,58,59]. In these structures, the H8 hydrogen atom loses its sigma bond with the C4 carbon atom, while a new C6-H8 sigma bond is formed.

**Figure 8:**
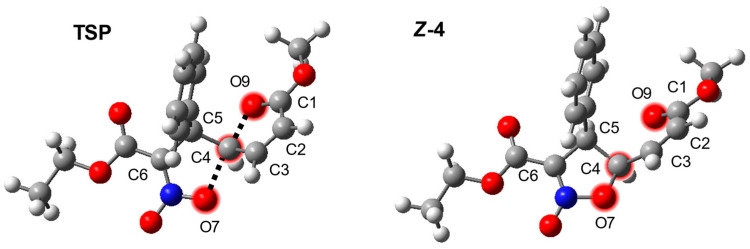
Views of key structures for the formation of the ***Z*-4** nitronate in the reaction between 2-methoxyfuran (**1**) and ethyl (Z)-3-phenyl-2-nitroprop-2-enoate (**2**) according to wb97xd/6-311+G(d,p) (PCM) calculations.

The final type of transition state in the context of this transformation is **TSP**. This structure was identified on the pathway leading to the five-membered nitronate ***Z*-4**. In this transition state, the C4-O9 bond is broken, while a new C4-C7 single bond is formed (Figure 8). Formally, this transition state can be treated as typical for intramolecular substitution at an sp^3^ carbon atom [60].

**Figure 9:**
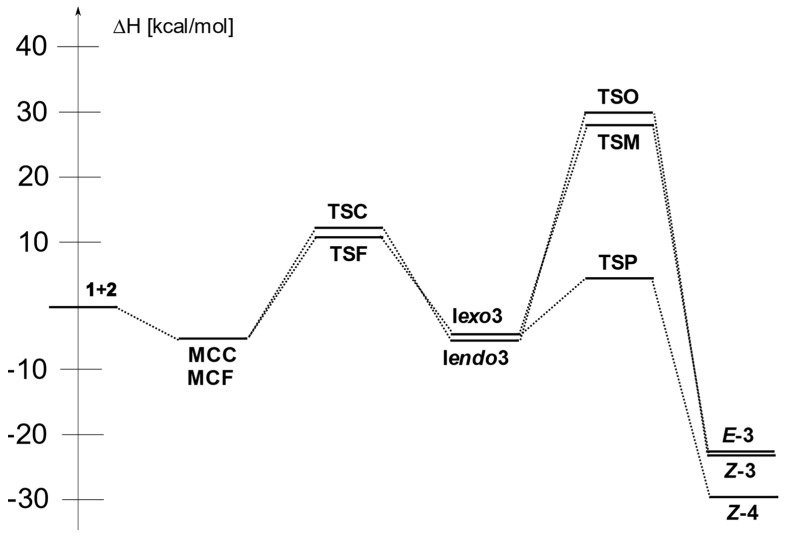
Enthalpy profiles for the formation of the ***Z*-3**, ***E*-3**, and ***Z*-4** adducts in the reaction between 2-methoxyfuran (**1**) and ethyl (Z)-3-phenyl-2-nitroprop-2-enoate (**2**) according to wb97xd/6-311+G(d,p) (PCM) calculations.

The enthalpy profiles realized in the practice paths leading to the adducts **3** and **4** are presented in Figure 9.

**Figure 10:**
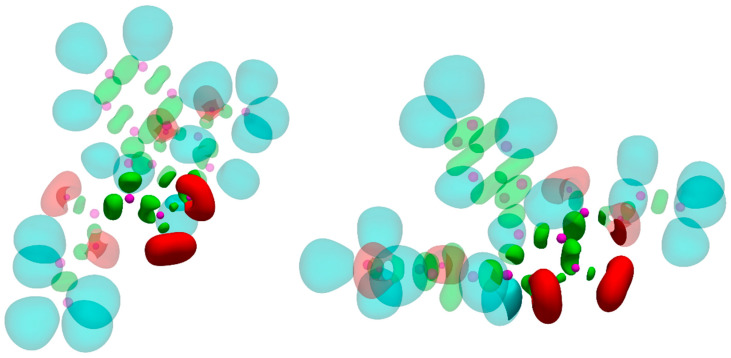
Topology of ***Z*-4** (**left**) and **HDA*exo*** (**right**) ELF, rendered at an isovalue of 0.8. Core basins are shown in magenta, protonated basins in cyan, disynaptic basins in green, and monosynaptic basins in red. Parts of the function, significant for intermediate type identification, are depicted as solid, while the rest are translucent.

**Figure 11:**
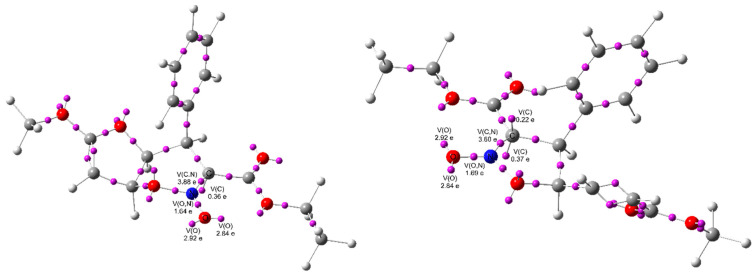
Positions and populations of significant ELF attractors of unprotonated basins in ***Z*–4** (**left**) and **HDA*exo*** (**right**).

**Scheme 6:**
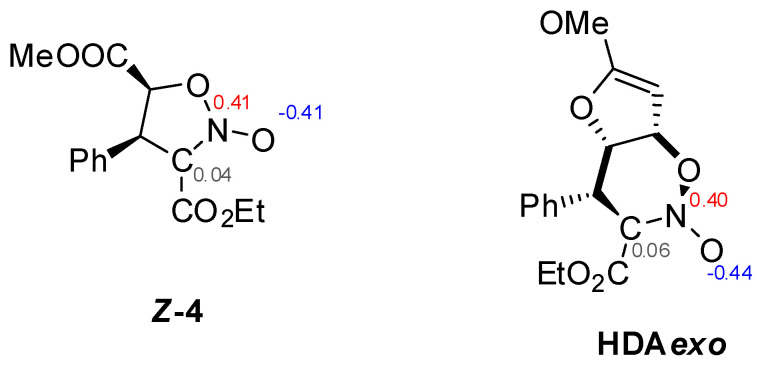
Natural charges of significant atoms in structures ***Z*–4** (**left**) and **HDA*exo*** (**right**), as computed using WB97XD/6-311+G(d,p) pcm CH_2_Cl_2_.

Finally, we also sought to explore the electronic nature of the localized six- and five-membered nitronates, as these compounds should be considered as potential three-atom components in [3+2] cycloaddition processes [61,62,63]. The five-membered nitronate ***Z*-4** features two nonreducible monosynaptic valence basins V(C), with populations of 0.37e and 0.22e, respectively (Figure 10). The charge on the C atom is negligible (Scheme 6). The disynaptic valence basin V(N,O) can be assigned as an underpopulated double bond, with a population of 3.60e (Figure 11). The N atom has a charge of 0.41e, while the O atom is negatively charged (−0.43e). The N–O bond is a slightly underpopulated single bond, and the O atom has a reducible monosynaptic basin with a total population of 5.76e, corresponding to three slightly underpopulated lone pairs. Based on the aforementioned computational results, the nitronate ***Z*-4** can be considered a predominantly zwitterionic type (zw-type) TAC [64].

An analogous analysis of the six-membered nitronate **HDA*exo*** reveals comparable results. The C atom has a negligible charge and a monosynaptic valence basin V(C), with a population of 0.36e. The C-N bonding basin has a population of 3.88e, indicating an underpopulated double bond. The N atom has a charge of 0.40e, while the oxygen atom is negatively charged (−0.44e). The V(N,O) disynaptic basin has a population of 1.64e, consistent with a slightly underpopulated single bond. These results allow us to classify the six-membered nitronate as a zw-type TAC as well [64].

## 3. Computational Details

The computational study was performed using the wb97xd/6-311+G(d,p) level of theory with the Gaussian package as the software [65]. The PlGrid infrastructure at the national computing center “Cyfronet” was utilized. A similar computational level and methodology have already been successfully applied to explore the mechanistic aspects of various cycloaddition processes, including Diels–Alder reactions, hydrogen shifts, and sigmatropic rearrangements. All localized stationary points were verified through a full vibrational analysis. We found that starting molecules, intermediates, and products had positive Hessian matrices, while all optimized transition states (**TSs**) exhibited only one negative eigenvalue in their Hessian matrices.

Next, intrinsic reaction coordinate (IRC) calculations were performed for all optimized transition states. The obtained IRC trajectories confirmed, without doubt, the postulated nature of the TSs and their role within the energy profile. The presence of solvent (dichloromethane) in the reaction environment was included using the IEFPCM (Integral Equation Formalism Polarizable Continuum Model) algorithm [66]. Calculations of all critical structures were performed at a temperature of T = 298 K and a pressure of p = 1 atm. The results of calculations are summarized in Table 2.

The global electron density transfer (GEDT) [67] within critical structures was estimated using the following formula:

GEDT = −ΣqA

where qA is the net charge, and the sum is taken over all of the atoms of nitroalkene.

The global and local electronic properties of the reactants were estimated using equations recommended by *Parr* and *Domingo* [40,68,69]. In particular, the electronic chemical potentials (µ) and chemical hardness (η) were evaluated in terms of the one-electron energies of the frontier molecular orbitals (HOMO and LUMO) using the following equations:

µ ≈ (E_HOMO_ + E_LUMO_)/2      η ≈ E_LUMO_ − E_HOMO_

The values of µ and η were then used to calculate the global electrophilicity index (ω) using the following formula:

ω = μ^2^/2η

Global nucleophilicity (N) [70] was expressed using the following equation:

N = E_HOMO_ − E_HOMO(tetracyanoethene)_

The local electrophilicity (ω_k_) at atom k was calculated by projecting the index ω onto any reaction center k in the molecule using *Parr* functions P^+^_k_ [71]:

ω_k_ = P^+^_k_·ω

The local nucleophilicity (N_k_) condensed to atom k was calculated using global nucleophilicity N and *Parr* functions P^−^_k_ [71] according to the following formula:

N_k_ = P^−^_k_·N

The results are summarized in Table 1.

## 4. Conclusions

Our WB97XD/6-311+G(d,p) (PCM) calculations clearly indicate that a fundamental revision of the view on the molecular mechanism of the reaction between 2-methoxyfuran and ethyl (*Z*)-3-phenyl-2-nitroprop-2-enoate is necessary. The first stage of the title transformation involves the formation of pre-reaction complexes. These initially formed complexes can further convert into respective zwitterions, a process driven by the nucleophilic attack of the nucleophilically activated 5-position of the furan on the electrophilically activated 2-position of the nitrovinyl moiety. It is important to emphasize that the zwitterionic nature of the optimized intermediates was confirmed by an ELF analysis of the electronic structure.

The final composition of the post-reaction mixture can vary depending on whether the reaction is under kinetic or thermodynamic control. In the thermodynamic scenario (as described in the experimental study of this reaction), the zwitterion is converted to the five-membered nitronate via intramolecular substitution at the sp^3^ carbon atom. Thus, the molecular mechanism and the reaction course of the addition of 2-methoxyfuran to ethyl (*Z*)-3-phenyl-2-nitroprop-2-enoate are completely different from those of typical processes with the participation of furan analogs and electrophilic alkenes. According to ELF and Natural Population Analyses (NPA), the nitronates ***Z*-4** and **HDA*exo*** are polar in nature and can be classified as zwitterionic-type TACs.

## Figures and Tables

**Table 1:** Global electronic properties of 2-methoxyfuran (**1**) and ethyl (*Z*)-3-phenyl-2-nitroprop-2-enoate (**2**).

	µ [eV]	η [eV]	ω [eV]	N [eV]
**1**	−2.23	6.34	0.39	3.72
**2**	−4.51	4.53	2.24	2.34

**Table 2:** Kinetic and thermodynamic parameters for key transformations in the reaction between 2-methoxyfuran (**1**) and ethyl (*Z*)-3-phenyl-2-nitroprop-2-enoate (**2**) based on wb97xd/6-311+G(d,p) (PCM) calculations (ΔH, ΔG are in kcal/mol; ΔS are in cal/molK).

Transformation	Transition	ΔH	ΔS	ΔG
The formation of intermediates	1+2 → MCA	−7.1	−40.3	4.9
	MCA → TSA	14.4	−11.8	18.0
	MCA → I*endo*1	1.7	−8.3	4.2
	1+2 → MCB	−4.5	−32.8	5.3
	MCB → TSB	16.5	−13.7	20.6
	MCB → I*endo*2	1.2	−15.2	5.7
	1+2 → MCC	−5.2	−38.2	6.2
	MCC → TSC	17.2	−8.4	19.7
	MCC → I*endo*3	−0.2	−7.5	2.0
	1+2 → MCD	−6.6	−39.7	5.3
	MCD → TSD	14.9	−9.7	17.8
	MCD → Ie*xo*1	4.7	−9.4	7.5
	1+2 → MCE	−4.4	−36.4	6.4
	MCE → TSE	17.3	−8.2	19.7
	MCE → I*exo*2	−0.7	−11.5	2.7
	1+2 → MCF	−5.4	−34.6	4.9
	MCF → TSF	16.5	−13.0	20.3
	MCF → I*exo*3	0.2	−15.2	4.7
The rotation around the nitroethyl moiety within intermediates	TS_rot(I*endo*1_ → _I*endo*2)_	4.8	−4.5	6.1
	I*endo*1 → I*endo*2	2.1	0.6	1.9
	TSrot_(I*endo*2_ → _I*endo*3)_	1.0	−4.9	2.5
	I*endo*2 → I*endo*3	−2.1	2.3	−2.8
	TS_rot(I*endo*3_ → I*_endo_*_1)_	3.5	−7.8	5.8
	I*endo*3 → I*endo*1	0.0	−2.9	0.8
	TS_rot(I*exo*1_ →_I*exo*2)_	0.9	−3.8	2.1
	I*exo*1 → I*exo*2	−3.2	0.8	−3.6
	TS_rot(I*exo*2_ → _I*exo*3)_	2.1	−4.9	3.6
	I*exo*2 → I*exo*3	−0.1	−2.0	0.5
The formation of hetero Diels–Alder adducts	I*exo*1 → TSG	1.4	−3.7	2.5
	I*exo*1 → HDA*exo*	−2.8	−3.8	−1.7
	I*exo*3 → TSG	4.8	−3.0	5.7
	I*exo*3 → HDA*exo*	0.5	−3.2	1.5
	I*endo*1 → TSI	0.7	−4.3	2.0
	I*endo*1 → HDA*endo*	−8.2	−2.4	−7.5
The formation of Diels–Alder adducts	I*endo*1 → TSJ	9.1	−8.0	11.5
	I*endo*1 → DA*endo*	−3.1	−7.9	−0.7
	I*exo*1 → TSK	10.6	−14.0	14.8
	I*exo*1 → DA*exo*	−3.9	−13.3	0.0
The formation of Michael adducts	I*exo*2 → TSL	32.5	0.5	32.3
	I*exo*2 → *Z*-3	−17.3	6.6	−19.3
	I*exo*3 → TSM	32.6	2.4	31.9
	I*exo*3 → *Z*-3	−17.2	8.6	−19.8
	I*endo*2 → TSN	33.4	1.8	32.9
	I*endo*2 → *E*-3	−19.6	1.5	−20.0
	I*endo*3 → TSO	35.5	−0.4	35.7
	I*endo*3 → *E*-3	−16.9	4.8	−18.4
The formation of nitronate ***Z*-4**	I*exo*3 → TSP	8.8	0.4	8.7
	I*exo*3 → *Z*-4	−26.2	4.7	−27.5

## Data Availability

Data are contained within the article.

## Supplementary Materials

The following supporting information can be downloaded at: https://www.mdpi.com/article/10.3390/molecules29204876/s1, cartesian coordinates for transition states.
