# Supplementary material for: A New Insight into the Molecular Mechanism of the Reaction between 2-Methoxyfuran and Ethyl (Z)-3-phenyl-2-nitroprop-2-enoate: An Molecular Electron Density Theory (MEDT) Computational Study
Source: Molecules. 2024 Oct 14;29(20):4876. doi: 10.3390/molecules29204876 (PMC11510463; doi:10.3390/molecules29204876)
Supplement: Supplementary file 1 [file molecules-29-04876-s001.zip › molecules-3252789-supplementary.pdf]

# SUPPLEMENTARY MATERIAL

## A new insight on the molecular mechanism of the reaction between 2-methoxyfuran and ethyl (Z)-3-phenyl-2-nitroprop-2-enoate: MEDT computational study and considerations

Mikołaj Sadowski[1], Ewa Dresler[2], Aneta Wróblewska[3], Radomir Jasiński[1]*

[1] Department of Organic Chemistry and Technology, Cracow University of Technology, Warszawska 24, 31-155 Krakow, Poland
[2] Łukasiewicz Research Network—Institute of Heavy Organic Synthesis “Blachownia”, Energetyków 9, 47-225 Kędzierzyn-Koźle, Poland
[3] Department of Organic Chemistry, University of Lodz, Tamka 12, 91-403 Łódź, Poland
* Correspondence: radomir.jasinski@pk.edu.pl

# CARTESIAN COORDINATES FOR TRANSITION STATES

**TSA**

| Center Number | Atomic Number | Atomic Type | Coordinates (Angstroms) X | Y | Z |
|---|---|---|---|---|---|
| 1 | 6 | 0 | -0.674021 | -1.927962 | -0.788408 |
| 2 | 6 | 0 | 1.460258 | -1.917437 | -0.371698 |
| 3 | 6 | 0 | -1.036994 | 0.107123 | -0.602044 |
| 4 | 1 | 0 | -0.843366 | 0.228567 | -1.663957 |
| 5 | 6 | 0 | 0.033221 | 0.676221 | 0.102830 |
| 6 | 8 | 0 | 0.588955 | -1.815341 | -1.360015 |
| 7 | 6 | 0 | 1.205483 | 1.136138 | -0.667619 |
| 8 | 8 | 0 | 1.173854 | 1.286688 | -1.873284 |
| 9 | 8 | 0 | 2.315927 | 1.297733 | 0.049793 |
| 10 | 7 | 0 | -0.031211 | 0.874549 | 1.507618 |
| 11 | 8 | 0 | -0.765905 | 0.130968 | 2.158639 |
| 12 | 8 | 0 | 0.604368 | 1.784992 | 2.029710 |
| 13 | 6 | 0 | -2.502183 | 0.214378 | -0.327808 |
| 14 | 6 | 0 | -3.234366 | 0.923795 | -1.288510 |
| 15 | 6 | 0 | -3.189673 | -0.338147 | 0.754026 |
| 16 | 6 | 0 | -4.604128 | 1.107551 | -1.155039 |
| 17 | 1 | 0 | -2.721735 | 1.346027 | -2.146605 |
| 18 | 6 | 0 | -4.563100 | -0.165667 | 0.880135 |
| 19 | 1 | 0 | -2.654014 | -0.898478 | 1.504077 |
| 20 | 6 | 0 | -5.274957 | 0.561017 | -0.066973 |
| 21 | 1 | 0 | -5.146040 | 1.670381 | -1.906389 |
| 22 | 1 | 0 | -5.077894 | -0.604542 | 1.727293 |
| 23 | 1 | 0 | -6.345698 | 0.693945 | 0.038073 |
| 24 | 6 | 0 | 3.489362 | 1.726205 | -0.666150 |
| 25 | 1 | 0 | 3.770743 | 0.941148 | -1.372377 |
| 26 | 1 | 0 | 3.247426 | 2.625728 | -1.235259 |
| 27 | 6 | 0 | 4.573803 | 1.986606 | 0.352942 |
| 28 | 1 | 0 | 4.806618 | 1.085072 | 0.923411 |
| 29 | 1 | 0 | 5.481836 | 2.309972 | -0.160949 |
| 30 | 1 | 0 | 4.272839 | 2.771781 | 1.049213 |
| 31 | 1 | 0 | -1.457448 | -2.176336 | -1.487018 |
| 32 | 8 | 0 | 2.700450 | -1.696316 | -0.716118 |
| 33 | 6 | 0 | 3.664089 | -1.736676 | 0.346987 |
| 34 | 1 | 0 | 4.618100 | -1.496946 | -0.113896 |
| 35 | 1 | 0 | 3.409107 | -0.989826 | 1.100920 |
| 36 | 1 | 0 | 3.699489 | -2.735624 | 0.785210 |
| 37 | 6 | 0 | 0.852527 | -2.281236 | 0.822940 |
| 38 | 6 | 0 | -0.503484 | -2.360856 | 0.527701 |
| 39 | 1 | 0 | 1.339286 | -2.439766 | 1.770088 |
| 40 | 1 | 0 | -1.296996 | -2.629059 | 1.205149 |

**TSB**

| Center Number | Atomic Number | Atomic Type | Coordinates (Angstroms) X | Y | Z |
|---|---|---|---|---|---|
| 1 | 6 | 0 | 0.456393 | -1.514686 | -0.683872 |
| 2 | 6 | 0 | 2.622047 | -1.702416 | -0.466084 |
| 3 | 6 | 0 | -0.244925 | 0.024887 | 0.572897 |
| 4 | 1 | 0 | -0.428906 | -0.577199 | 1.455555 |
| 5 | 6 | 0 | -1.500119 | 0.414489 | 0.048168 |
| 6 | 8 | 0 | 1.688221 | -1.113069 | -1.191118 |
| 7 | 6 | 0 | -2.684817 | -0.165338 | 0.685665 |
| 8 | 8 | 0 | -2.615411 | -0.811173 | 1.719859 |
| 9 | 8 | 0 | -3.832837 | 0.010301 | 0.032740 |
| 10 | 7 | 0 | -1.599015 | 1.114290 | -1.171815 |
| 11 | 8 | 0 | -2.574431 | 1.819096 | -1.414316 |
| 12 | 8 | 0 | -0.666058 | 0.992750 | -1.981074 |
| 13 | 6 | 0 | 1.002548 | 0.802396 | 0.730030 |
| 14 | 6 | 0 | 1.863336 | 0.391756 | 1.756951 |
| 15 | 6 | 0 | 1.365063 | 1.921536 | -0.032694 |
| 16 | 6 | 0 | 3.054047 | 1.063840 | 2.011059 |
| 17 | 1 | 0 | 1.587609 | -0.454300 | 2.376071 |
| 18 | 6 | 0 | 2.541564 | 2.602682 | 0.237004 |
| 19 | 1 | 0 | 0.734598 | 2.256580 | -0.843049 |
| 20 | 6 | 0 | 3.396219 | 2.172492 | 1.250815 |
| 21 | 1 | 0 | 3.703923 | 0.723432 | 2.808634 |
| 22 | 1 | 0 | 2.800241 | 3.472613 | -0.355845 |
| 23 | 1 | 0 | 4.319882 | 2.705110 | 1.446495 |
| 24 | 6 | 0 | -5.007956 | -0.572892 | 0.623077 |
| 25 | 1 | 0 | -4.867411 | -1.653618 | 0.699759 |
| 26 | 1 | 0 | -5.131034 | -0.173615 | 1.632205 |
| 27 | 6 | 0 | -6.179590 | -0.222443 | -0.264310 |
| 28 | 1 | 0 | -6.041644 | -0.624342 | -1.270320 |
| 29 | 1 | 0 | -7.092816 | -0.650550 | 0.155554 |
| 30 | 1 | 0 | -6.305955 | 0.859863 | -0.333746 |
| 31 | 1 | 0 | -0.338292 | -1.448090 | -1.406675 |
| 32 | 8 | 0 | 3.871540 | -1.400999 | -0.660775 |
| 33 | 6 | 0 | 4.175211 | -0.365887 | -1.618569 |
| 34 | 1 | 0 | 5.252850 | -0.243129 | -1.569496 |
| 35 | 1 | 0 | 3.869937 | -0.680775 | -2.616393 |
| 36 | 1 | 0 | 3.667738 | 0.556804 | -1.336334 |
| 37 | 6 | 0 | 2.082593 | -2.599701 | 0.449096 |
| 38 | 6 | 0 | 0.710046 | -2.515363 | 0.262974 |
| 39 | 1 | 0 | -0.056054 | -3.062801 | 0.791387 |
| 40 | 1 | 0 | 2.640291 | -3.193376 | 1.153134 |

**TSC**

| Center Number | Atomic Number | Atomic Type | Coordinates (Angstroms) X | Y | Z |
|---|---|---|---|---|---|
| 1 | 6 | 0 | 0.456256 | -1.515151 | -0.683181 |
| 2 | 6 | 0 | 2.622021 | -1.702412 | -0.466128 |
| 3 | 6 | 0 | -0.244895 | 0.024879 | 0.573138 |
| 4 | 1 | 0 | -0.428927 | -0.576997 | 1.455928 |
| 5 | 6 | 0 | -1.500040 | 0.414493 | 0.048315 |
| 6 | 8 | 0 | 1.687832 | -1.113474 | -1.191013 |
| 7 | 6 | 0 | -2.684809 | -0.165235 | 0.685757 |
| 8 | 8 | 0 | -2.615584 | -0.810650 | 1.720225 |
| 9 | 8 | 0 | -3.832659 | 0.009960 | 0.032412 |
| 10 | 7 | 0 | -1.598912 | 1.114223 | -1.171713 |
| 11 | 8 | 0 | -2.574267 | 1.819147 | -1.414146 |
| 12 | 8 | 0 | -0.666044 | 0.992544 | -1.981035 |
| 13 | 6 | 0 | 1.002615 | 0.802375 | 0.730092 |
| 14 | 6 | 0 | 1.863391 | 0.391972 | 1.757117 |
| 15 | 6 | 0 | 1.365116 | 1.921382 | -0.032837 |
| 16 | 6 | 0 | 3.054057 | 1.064166 | 2.011145 |
| 17 | 1 | 0 | 1.587671 | -0.453958 | 2.376408 |
| 18 | 6 | 0 | 2.541573 | 2.602639 | 0.236775 |
| 19 | 1 | 0 | 0.734661 | 2.256240 | -0.843278 |
| 20 | 6 | 0 | 3.396205 | 2.172691 | 1.250707 |
| 21 | 1 | 0 | 3.703915 | 0.723944 | 2.808815 |
| 22 | 1 | 0 | 2.800228 | 3.472469 | -0.356231 |
| 23 | 1 | 0 | 4.319831 | 2.705396 | 1.446327 |
| 24 | 6 | 0 | -5.007922 | -0.572974 | 0.622709 |
| 25 | 1 | 0 | -4.867616 | -1.653737 | 0.699345 |
| 26 | 1 | 0 | -5.130938 | -0.173715 | 1.631847 |
| 27 | 6 | 0 | -6.179456 | -0.222245 | -0.264703 |
| 28 | 1 | 0 | -6.041483 | -0.623995 | -1.270768 |
| 29 | 1 | 0 | -7.092762 | -0.650315 | 0.155024 |
| 30 | 1 | 0 | -6.305690 | 0.860087 | -0.333974 |
| 31 | 1 | 0 | -0.338680 | -1.449212 | -1.405761 |
| 32 | 8 | 0 | 3.871388 | -1.400837 | -0.661358 |
| 33 | 6 | 0 | 4.174539 | -0.365925 | -1.619534 |
| 34 | 1 | 0 | 5.252175 | -0.242975 | -1.570886 |
| 35 | 1 | 0 | 3.868949 | -0.681121 | -2.617164 |
| 36 | 1 | 0 | 3.667011 | 0.556750 | -1.337349 |
| 37 | 6 | 0 | 2.083064 | -2.599511 | 0.449542 |
| 38 | 6 | 0 | 0.710445 | -2.515505 | 0.263877 |
| 39 | 1 | 0 | -0.055376 | -3.062923 | 0.792717 |
| 40 | 1 | 0 | 2.641136 | -3.192846 | 1.153571 |

**TSD**

| Center Number | Atomic Number | Atomic Type | Coordinates (Angstroms) X | Y | Z |
|---|---|---|---|---|---|
| 1 | 6 | 0 | -0.120695 | -0.699751 | -0.084880 |
| 2 | 6 | 0 | -1.015157 | 2.054772 | 0.250127 |
| 3 | 6 | 0 | 0.694311 | 1.504135 | 1.469963 |
| 4 | 6 | 0 | 0.988089 | -0.393528 | 0.725787 |
| 5 | 1 | 0 | 0.883003 | -0.891401 | 1.683172 |
| 6 | 8 | 0 | 0.299226 | 2.136813 | 0.296069 |
| 7 | 7 | 0 | -0.076025 | -0.575995 | -1.503554 |
| 8 | 8 | 0 | 0.620474 | 0.307121 | -1.995969 |
| 9 | 8 | 0 | -0.699443 | -1.379228 | -2.192638 |
| 10 | 6 | 0 | 2.418271 | -0.363562 | 0.288275 |
| 11 | 6 | 0 | 3.219999 | -1.415791 | 0.742132 |
| 12 | 6 | 0 | 2.999179 | 0.632192 | -0.498513 |
| 13 | 6 | 0 | 4.562098 | -1.491127 | 0.391152 |
| 14 | 1 | 0 | 2.785723 | -2.187826 | 1.368898 |
| 15 | 6 | 0 | 4.345639 | 0.564575 | -0.834672 |
| 16 | 1 | 0 | 2.397051 | 1.458960 | -0.846826 |
| 17 | 6 | 0 | 5.129791 | -0.498145 | -0.399037 |
| 18 | 1 | 0 | 5.163774 | -2.321358 | 0.743042 |
| 19 | 1 | 0 | 4.782579 | 1.347723 | -1.443876 |
| 20 | 1 | 0 | 6.178701 | -0.548420 | -0.668502 |
| 21 | 6 | 0 | -1.293765 | -1.294764 | 0.571237 |
| 22 | 8 | 0 | -1.259385 | -1.685633 | 1.723848 |
| 23 | 8 | 0 | -2.413435 | -1.287360 | -0.152890 |
| 24 | 6 | 0 | -3.595238 | -1.804714 | 0.482957 |
| 25 | 1 | 0 | -3.398704 | -2.824541 | 0.819866 |
| 26 | 1 | 0 | -3.817764 | -1.193548 | 1.361616 |
| 27 | 6 | 0 | -4.712989 | -1.756953 | -0.532357 |
| 28 | 1 | 0 | -4.471020 | -2.365155 | -1.406017 |
| 29 | 1 | 0 | -5.628307 | -2.147470 | -0.081960 |
| 30 | 1 | 0 | -4.903856 | -0.733015 | -0.861291 |
| 31 | 1 | 0 | 1.692128 | 1.756234 | 1.792642 |
| 32 | 8 | 0 | -1.553993 | 2.459247 | -0.865858 |
| 33 | 6 | 0 | -2.970133 | 2.251864 | -0.993048 |
| 34 | 1 | 0 | -3.225557 | 2.587794 | -1.993444 |
| 35 | 1 | 0 | -3.193756 | 1.189331 | -0.881275 |
| 36 | 1 | 0 | -3.505607 | 2.844234 | -0.249173 |
| 37 | 6 | 0 | -1.548614 | 1.598848 | 1.456562 |
| 38 | 6 | 0 | -0.451085 | 1.323108 | 2.255438 |
| 39 | 1 | 0 | -2.592828 | 1.466303 | 1.685023 |
| 40 | 1 | 0 | -0.459139 | 0.919502 | 3.255754 |

**TSE**

| Center Number | Atomic Number | Atomic Type | Coordinates (Angstroms) X | Y | Z |
|---|---|---|---|---|---|
| 1 | 6 | 0 | -0.225170 | -1.013727 | 0.056124 |
| 2 | 6 | 0 | -0.845074 | 2.900414 | -0.212189 |
| 3 | 6 | 0 | 0.567864 | 1.470285 | -1.077906 |
| 4 | 6 | 0 | 0.773548 | -0.072274 | 0.314321 |
| 5 | 1 | 0 | 0.465210 | 0.560316 | 1.142947 |
| 6 | 8 | 0 | -0.781832 | 1.792792 | -0.932747 |
| 7 | 7 | 0 | -0.026781 | -2.043678 | -0.906663 |
| 8 | 8 | 0 | -0.490518 | -3.159666 | -0.714629 |
| 9 | 8 | 0 | 0.632674 | -1.765623 | -1.911058 |
| 10 | 6 | 0 | 2.245818 | -0.318689 | 0.314848 |
| 11 | 6 | 0 | 2.869207 | -0.250859 | 1.565841 |
| 12 | 6 | 0 | 3.029577 | -0.568793 | -0.812344 |
| 13 | 6 | 0 | 4.235879 | -0.460037 | 1.692395 |
| 14 | 1 | 0 | 2.275561 | -0.038807 | 2.448897 |
| 15 | 6 | 0 | 4.400140 | -0.764796 | -0.684845 |
| 16 | 1 | 0 | 2.573483 | -0.608487 | -1.790906 |
| 17 | 6 | 0 | 5.007358 | -0.717285 | 0.564352 |
| 18 | 1 | 0 | 4.697628 | -0.413441 | 2.671999 |
| 19 | 1 | 0 | 4.994876 | -0.954455 | -1.571128 |
| 20 | 1 | 0 | 6.076135 | -0.872484 | 0.657890 |
| 21 | 6 | 0 | -1.533997 | -0.838976 | 0.691044 |
| 22 | 8 | 0 | -1.740811 | 0.000799 | 1.550189 |
| 23 | 8 | 0 | -2.491570 | -1.630711 | 0.212757 |
| 24 | 6 | 0 | -3.795039 | -1.510813 | 0.810448 |
| 25 | 1 | 0 | -3.705774 | -1.682893 | 1.885106 |
| 26 | 1 | 0 | -4.163691 | -0.493482 | 0.658844 |
| 27 | 6 | 0 | -4.690918 | -2.531739 | 0.149743 |
| 28 | 1 | 0 | -4.310666 | -3.542783 | 0.307522 |
| 29 | 1 | 0 | -5.692240 | -2.467863 | 0.581622 |
| 30 | 1 | 0 | -4.766235 | -2.348587 | -0.924141 |
| 31 | 1 | 0 | 0.782618 | 0.860348 | -1.941631 |
| 32 | 8 | 0 | -2.006491 | 3.360050 | 0.158876 |
| 33 | 6 | 0 | -3.185158 | 2.625923 | -0.227685 |
| 34 | 1 | 0 | -4.017172 | 3.196059 | 0.174746 |
| 35 | 1 | 0 | -3.251648 | 2.569241 | -1.314366 |
| 36 | 1 | 0 | -3.153662 | 1.629696 | 0.211537 |
| 37 | 6 | 0 | 0.412804 | 3.408716 | 0.071262 |
| 38 | 6 | 0 | 1.301526 | 2.520818 | -0.530664 |
| 39 | 1 | 0 | 2.379722 | 2.574956 | -0.523893 |
| 40 | 1 | 0 | 0.621492 | 4.288277 | 0.656024 |

**TSF**

| Center Number | Atomic Number | Atomic Type | Coordinates (Angstroms) X | Y | Z |
|---|---|---|---|---|---|
| 1 | 6 | 0 | -1.504207 | 0.442095 | -0.120591 |
| 2 | 6 | 0 | 2.569245 | -1.503632 | 0.856851 |
| 3 | 6 | 0 | 0.417421 | -1.630625 | 0.498621 |
| 4 | 6 | 0 | -0.195007 | 0.103717 | -0.517483 |
| 5 | 1 | 0 | -0.278257 | -0.499915 | -1.415665 |
| 6 | 8 | 0 | 1.677154 | -1.895554 | -0.039135 |
| 7 | 7 | 0 | -1.775612 | 1.326903 | 0.946577 |
| 8 | 8 | 0 | -2.793915 | 2.010852 | 0.938792 |
| 9 | 8 | 0 | -0.955783 | 1.391326 | 1.871310 |
| 10 | 6 | 0 | 1.053176 | 0.899760 | -0.552587 |
| 11 | 6 | 0 | 1.793669 | 0.799893 | -1.742097 |
| 12 | 6 | 0 | 1.568039 | 1.695214 | 0.478172 |
| 13 | 6 | 0 | 2.980083 | 1.496065 | -1.913977 |
| 14 | 1 | 0 | 1.424870 | 0.172052 | -2.546499 |
| 15 | 6 | 0 | 2.761615 | 2.386720 | 0.306538 |
| 16 | 1 | 0 | 1.039221 | 1.762146 | 1.415384 |
| 17 | 6 | 0 | 3.470107 | 2.293397 | -0.884470 |
| 18 | 1 | 0 | 3.524464 | 1.410476 | -2.847371 |
| 19 | 1 | 0 | 3.142570 | 2.995834 | 1.118214 |
| 20 | 1 | 0 | 4.402024 | 2.833100 | -1.009069 |
| 21 | 6 | 0 | -2.589595 | -0.315104 | -0.752983 |
| 22 | 8 | 0 | -2.379890 | -1.119896 | -1.647157 |
| 23 | 8 | 0 | -3.802102 | -0.121846 | -0.238523 |
| 24 | 6 | 0 | -4.874939 | -0.896363 | -0.804540 |
| 25 | 1 | 0 | -4.937039 | -0.682750 | -1.873749 |
| 26 | 1 | 0 | -4.648381 | -1.957965 | -0.681817 |
| 27 | 6 | 0 | -6.142722 | -0.506817 | -0.081126 |
| 28 | 1 | 0 | -6.354110 | 0.556525 | -0.209943 |
| 29 | 1 | 0 | -6.981162 | -1.077095 | -0.487676 |
| 30 | 1 | 0 | -6.065429 | -0.722159 | 0.986685 |
| 31 | 1 | 0 | -0.367597 | -2.246339 | 0.087950 |
| 32 | 8 | 0 | 3.836534 | -1.519980 | 0.553037 |
| 33 | 6 | 0 | 4.212947 | -1.937958 | -0.771881 |
| 34 | 1 | 0 | 5.295572 | -1.857909 | -0.797511 |
| 35 | 1 | 0 | 3.901938 | -2.968677 | -0.943436 |
| 36 | 1 | 0 | 3.762345 | -1.273847 | -1.510176 |
| 37 | 6 | 0 | 1.974068 | -1.098142 | 2.041701 |
| 38 | 6 | 0 | 0.607668 | -1.224358 | 1.820340 |
| 39 | 1 | 0 | -0.187605 | -0.982452 | 2.506758 |
| 40 | 1 | 0 | 2.490479 | -0.731729 | 2.912361 |

**TSG**

| Center Number | Atomic Number | Atomic Type | Coordinates (Angstroms) X | Y | Z |
|---|---|---|---|---|---|
| 1 | 6 | 0 | -1.294162 | -0.338091 | -0.036289 |
| 2 | 6 | 0 | 3.041375 | -1.238766 | -0.571292 |
| 3 | 6 | 0 | 0.884022 | -1.046916 | -1.083585 |
| 4 | 6 | 0 | -0.079089 | 0.133108 | -0.791389 |
| 5 | 1 | 0 | -0.463905 | 0.437964 | -1.763052 |
| 6 | 8 | 0 | 2.224847 | -0.564820 | -1.341239 |
| 7 | 7 | 0 | -1.115544 | -0.902858 | 1.172426 |
| 8 | 8 | 0 | -2.017195 | -1.254896 | 1.945953 |
| 9 | 8 | 0 | 0.114188 | -1.112348 | 1.531976 |
| 10 | 6 | 0 | 0.554755 | 1.386704 | -0.193644 |
| 11 | 6 | 0 | 0.154238 | 2.610303 | -0.735013 |
| 12 | 6 | 0 | 1.476085 | 1.396286 | 0.856086 |
| 13 | 6 | 0 | 0.648268 | 3.811948 | -0.241004 |
| 14 | 1 | 0 | -0.559021 | 2.621807 | -1.553069 |
| 15 | 6 | 0 | 1.978222 | 2.597252 | 1.344904 |
| 16 | 1 | 0 | 1.790403 | 0.468248 | 1.311793 |
| 17 | 6 | 0 | 1.566401 | 3.809338 | 0.802422 |
| 18 | 1 | 0 | 0.318649 | 4.748504 | -0.676860 |
| 19 | 1 | 0 | 2.693944 | 2.582655 | 2.159569 |
| 20 | 1 | 0 | 1.959084 | 4.743205 | 1.188726 |
| 21 | 6 | 0 | -2.599214 | -0.105681 | -0.654905 |
| 22 | 8 | 0 | -2.697757 | 0.399175 | -1.763019 |
| 23 | 8 | 0 | -3.662284 | -0.477352 | 0.053284 |
| 24 | 6 | 0 | -4.947278 | -0.250248 | -0.553770 |
| 25 | 1 | 0 | -5.051398 | 0.814793 | -0.772439 |
| 26 | 1 | 0 | -4.993982 | -0.796845 | -1.498426 |
| 27 | 6 | 0 | -5.996721 | -0.727102 | 0.423161 |
| 28 | 1 | 0 | -5.934819 | -0.173633 | 1.362360 |
| 29 | 1 | 0 | -6.989245 | -0.569723 | -0.005455 |
| 30 | 1 | 0 | -5.877580 | -1.791578 | 0.635658 |
| 31 | 1 | 0 | 0.561363 | -1.542510 | -2.002435 |
| 32 | 8 | 0 | 4.282508 | -0.917105 | -0.703588 |
| 33 | 6 | 0 | 5.242431 | -1.578572 | 0.147936 |
| 34 | 1 | 0 | 6.198499 | -1.127188 | -0.096657 |
| 35 | 1 | 0 | 4.992349 | -1.398279 | 1.193457 |
| 36 | 1 | 0 | 5.258093 | -2.645387 | -0.074989 |
| 37 | 6 | 0 | 2.419731 | -2.199286 | 0.229851 |
| 38 | 6 | 0 | 1.069550 | -2.076593 | -0.019263 |
| 39 | 1 | 0 | 2.910510 | -2.895540 | 0.887683 |
| 40 | 1 | 0 | 0.329736 | -2.839818 | 0.164983 |

**TSI**

| Center Number | Atomic Number | Atomic Type | Coordinates (Angstroms) X | Y | Z |
|---|---|---|---|---|---|
| 1 | 6 | 0 | -0.483807 | -1.691113 | -0.730914 |
| 2 | 6 | 0 | 1.630062 | -2.312634 | -0.423417 |
| 3 | 6 | 0 | -0.877608 | -0.201311 | -0.758109 |
| 4 | 1 | 0 | -0.722502 | 0.120829 | -1.790170 |
| 5 | 6 | 0 | 0.085065 | 0.603042 | 0.068529 |
| 6 | 8 | 0 | 0.829864 | -1.801841 | -1.328732 |
| 7 | 6 | 0 | 0.937742 | 1.572929 | -0.613160 |
| 8 | 8 | 0 | 0.811430 | 1.799613 | -1.806323 |
| 9 | 8 | 0 | 1.861962 | 2.173092 | 0.133508 |
| 10 | 7 | 0 | 0.119349 | 0.347615 | 1.388983 |
| 11 | 8 | 0 | -0.623968 | -0.633890 | 1.792716 |
| 12 | 8 | 0 | 0.793858 | 0.957001 | 2.229922 |
| 13 | 6 | 0 | -2.344039 | 0.050038 | -0.424009 |
| 14 | 6 | 0 | -2.764065 | 1.376440 | -0.301869 |
| 15 | 6 | 0 | -3.293285 | -0.959970 | -0.285805 |
| 16 | 6 | 0 | -4.091972 | 1.684762 | -0.042005 |
| 17 | 1 | 0 | -2.039528 | 2.176708 | -0.409460 |
| 18 | 6 | 0 | -4.626188 | -0.653906 | -0.023137 |
| 19 | 1 | 0 | -3.019354 | -2.005032 | -0.370124 |
| 20 | 6 | 0 | -5.031497 | 0.667883 | 0.100372 |
| 21 | 1 | 0 | -4.393805 | 2.722141 | 0.049280 |
| 22 | 1 | 0 | -5.345837 | -1.457222 | 0.088089 |
| 23 | 1 | 0 | -6.068737 | 0.905414 | 0.307137 |
| 24 | 6 | 0 | 2.714488 | 3.116364 | -0.540990 |
| 25 | 1 | 0 | 3.236030 | 2.604661 | -1.353033 |
| 26 | 1 | 0 | 2.094969 | 3.902735 | -0.977870 |
| 27 | 6 | 0 | 3.677634 | 3.666989 | 0.484613 |
| 28 | 1 | 0 | 4.286487 | 2.869667 | 0.916061 |
| 29 | 1 | 0 | 4.343579 | 4.388553 | 0.005629 |
| 30 | 1 | 0 | 3.142429 | 4.173630 | 1.290259 |
| 31 | 1 | 0 | -1.150517 | -2.289851 | -1.353077 |
| 32 | 8 | 0 | 2.854492 | -2.438417 | -0.810440 |
| 33 | 6 | 0 | 3.807231 | -2.957992 | 0.140691 |
| 34 | 1 | 0 | 4.764095 | -2.931854 | -0.370726 |
| 35 | 1 | 0 | 3.830266 | -2.320238 | 1.024399 |
| 36 | 1 | 0 | 3.544738 | -3.983269 | 0.402144 |
| 37 | 6 | 0 | 1.000664 | -2.670373 | 0.767298 |
| 38 | 6 | 0 | -0.318684 | -2.278735 | 0.636192 |
| 39 | 1 | 0 | 1.461157 | -3.156783 | 1.609860 |
| 40 | 1 | 0 | -1.150464 | -2.672334 | 1.197818 |

**TSJ**

| Center Number | Atomic Number | Atomic Type | Coordinates (Angstroms) X | Y | Z |
|---|---|---|---|---|---|
| 1 | 6 | 0 | -0.820658 | -1.822668 | -1.069169 |
| 2 | 6 | 0 | 1.183518 | -1.764867 | -0.306720 |
| 3 | 6 | 0 | -0.957608 | -0.264253 | -0.825546 |
| 4 | 1 | 0 | -0.758448 | 0.156178 | -1.812783 |
| 5 | 6 | 0 | 0.254685 | 0.114444 | -0.003173 |
| 6 | 8 | 0 | 0.553259 | -1.888144 | -1.482432 |
| 7 | 6 | 0 | 1.238571 | 1.004377 | -0.691420 |
| 8 | 8 | 0 | 1.075783 | 1.372493 | -1.836831 |
| 9 | 8 | 0 | 2.339012 | 1.263731 | -0.001962 |
| 10 | 7 | 0 | 0.039440 | 0.281110 | 1.413283 |
| 11 | 8 | 0 | -0.959199 | -0.250583 | 1.889010 |
| 12 | 8 | 0 | 0.847292 | 0.887876 | 2.108512 |
| 13 | 6 | 0 | -2.319744 | 0.248454 | -0.416431 |
| 14 | 6 | 0 | -2.482890 | 1.621451 | -0.219399 |
| 15 | 6 | 0 | -3.435638 | -0.576290 | -0.308151 |
| 16 | 6 | 0 | -3.724011 | 2.152347 | 0.102572 |
| 17 | 1 | 0 | -1.626190 | 2.281307 | -0.315883 |
| 18 | 6 | 0 | -4.683343 | -0.046155 | 0.008447 |
| 19 | 1 | 0 | -3.352838 | -1.645708 | -0.465045 |
| 20 | 6 | 0 | -4.832305 | 1.318265 | 0.219626 |
| 21 | 1 | 0 | -3.827068 | 3.220216 | 0.259630 |
| 22 | 1 | 0 | -5.539268 | -0.706572 | 0.091818 |
| 23 | 1 | 0 | -5.802940 | 1.730321 | 0.470942 |
| 24 | 6 | 0 | 3.361789 | 2.022437 | -0.676581 |
| 25 | 1 | 0 | 3.654513 | 1.484544 | -1.580952 |
| 26 | 1 | 0 | 2.945794 | 2.987937 | -0.971540 |
| 27 | 6 | 0 | 4.514360 | 2.176839 | 0.287153 |
| 28 | 1 | 0 | 4.917047 | 1.203275 | 0.574114 |
| 29 | 1 | 0 | 5.311426 | 2.750440 | -0.191064 |
| 30 | 1 | 0 | 4.200824 | 2.706382 | 1.188916 |
| 31 | 1 | 0 | -1.461735 | -2.185840 | -1.866539 |
| 32 | 8 | 0 | 2.481975 | -1.629084 | -0.415298 |
| 33 | 6 | 0 | 3.241191 | -1.641281 | 0.805730 |
| 34 | 1 | 0 | 4.262137 | -1.413941 | 0.512767 |
| 35 | 1 | 0 | 2.868642 | -0.870921 | 1.481518 |
| 36 | 1 | 0 | 3.198299 | -2.629882 | 1.265104 |
| 37 | 6 | 0 | 0.429649 | -2.512308 | 0.704905 |
| 38 | 6 | 0 | -0.814061 | -2.579470 | 0.220395 |
| 39 | 1 | 0 | 0.794887 | -2.811212 | 1.674454 |
| 40 | 1 | 0 | -1.687166 | -2.982537 | 0.710143 |

**TSK**

| Center Number | Atomic Number | Atomic Type | Coordinates (Angstroms) X | Y | Z |
|---|---|---|---|---|---|
| 1 | 6 | 0 | -0.208444 | -0.288998 | 0.012217 |
| 2 | 6 | 0 | -0.953061 | 1.824974 | 0.008783 |
| 3 | 6 | 0 | 0.693503 | 1.572346 | 1.368287 |
| 4 | 6 | 0 | 0.940854 | 0.062921 | 0.925326 |
| 5 | 1 | 0 | 0.807747 | -0.544599 | 1.820496 |
| 6 | 8 | 0 | 0.303721 | 2.217013 | 0.150578 |
| 7 | 7 | 0 | 0.030991 | -0.425797 | -1.380633 |
| 8 | 8 | 0 | 0.876010 | 0.298855 | -1.899621 |
| 9 | 8 | 0 | -0.623421 | -1.239325 | -2.037582 |
| 10 | 6 | 0 | 2.346692 | -0.213997 | 0.429725 |
| 11 | 6 | 0 | 2.798933 | -1.533271 | 0.475903 |
| 12 | 6 | 0 | 3.207507 | 0.772880 | -0.043349 |
| 13 | 6 | 0 | 4.081924 | -1.861795 | 0.055452 |
| 14 | 1 | 0 | 2.136598 | -2.310693 | 0.844649 |
| 15 | 6 | 0 | 4.495521 | 0.449155 | -0.454802 |
| 16 | 1 | 0 | 2.872695 | 1.800534 | -0.119766 |
| 17 | 6 | 0 | 4.937373 | -0.868327 | -0.408687 |
| 18 | 1 | 0 | 4.414374 | -2.893021 | 0.096427 |
| 19 | 1 | 0 | 5.151828 | 1.229920 | -0.822743 |
| 20 | 1 | 0 | 5.940714 | -1.119396 | -0.733780 |
| 21 | 6 | 0 | -1.251828 | -1.162479 | 0.612791 |
| 22 | 8 | 0 | -1.092835 | -1.763056 | 1.655440 |
| 23 | 8 | 0 | -2.421435 | -1.124306 | -0.021845 |
| 24 | 6 | 0 | -3.483379 | -1.938463 | 0.511574 |
| 25 | 1 | 0 | -3.163810 | -2.982571 | 0.494259 |
| 26 | 1 | 0 | -3.659194 | -1.650938 | 1.550363 |
| 27 | 6 | 0 | -4.702978 | -1.710192 | -0.350083 |
| 28 | 1 | 0 | -4.502506 | -1.979725 | -1.388841 |
| 29 | 1 | 0 | -5.525058 | -2.329756 | 0.014768 |
| 30 | 1 | 0 | -5.021304 | -0.665782 | -0.311930 |
| 31 | 1 | 0 | 1.576033 | 2.052517 | 1.778024 |
| 32 | 8 | 0 | -1.486707 | 2.122601 | -1.137525 |
| 33 | 6 | 0 | -2.898852 | 1.885942 | -1.305029 |
| 34 | 1 | 0 | -3.107448 | 2.131724 | -2.341722 |
| 35 | 1 | 0 | -3.116052 | 0.836684 | -1.110003 |
| 36 | 1 | 0 | -3.466439 | 2.541115 | -0.643010 |
| 37 | 6 | 0 | -1.577954 | 1.694776 | 1.327944 |
| 38 | 6 | 0 | -0.553944 | 1.605036 | 2.183959 |
| 39 | 1 | 0 | -2.636503 | 1.607437 | 1.514551 |
| 40 | 1 | 0 | -0.592490 | 1.419839 | 3.247052 |

**TSL**

| Center Number | Atomic Number | Atomic Type | Coordinates (Angstroms) X | Y | Z |
|---|---|---|---|---|---|
| 1 | 6 | 0 | -1.070797 | -0.034420 | 0.300605 |
| 2 | 6 | 0 | 2.779046 | -2.210663 | 0.314338 |
| 3 | 6 | 0 | 1.155722 | -0.726878 | 0.416662 |
| 4 | 6 | 0 | 0.296815 | 0.285159 | -0.328778 |
| 5 | 1 | 0 | 0.209319 | -0.118369 | -1.341447 |
| 6 | 8 | 0 | 1.809168 | -1.677920 | -0.399186 |
| 7 | 7 | 0 | -1.364374 | 0.612825 | 1.529653 |
| 8 | 8 | 0 | -2.511690 | 0.683786 | 1.959413 |
| 9 | 8 | 0 | -0.399614 | 1.060223 | 2.163611 |
| 10 | 6 | 0 | 0.748263 | 1.722678 | -0.433239 |
| 11 | 6 | 0 | 2.085730 | 2.079556 | -0.285434 |
| 12 | 6 | 0 | -0.183071 | 2.710956 | -0.750692 |
| 13 | 6 | 0 | 2.486386 | 3.402311 | -0.444470 |
| 14 | 1 | 0 | 2.826695 | 1.325891 | -0.040438 |
| 15 | 6 | 0 | 0.214386 | 4.032910 | -0.906505 |
| 16 | 1 | 0 | -1.228477 | 2.447471 | -0.875352 |
| 17 | 6 | 0 | 1.551951 | 4.383845 | -0.752857 |
| 18 | 1 | 0 | 3.531422 | 3.664478 | -0.322856 |
| 19 | 1 | 0 | -0.523061 | 4.790331 | -1.147219 |
| 20 | 1 | 0 | 1.862632 | 5.415648 | -0.871111 |
| 21 | 6 | 0 | -2.153837 | -0.429717 | -0.627778 |
| 22 | 8 | 0 | -2.008288 | -0.386504 | -1.835065 |
| 23 | 8 | 0 | -3.239013 | -0.924288 | -0.046196 |
| 24 | 6 | 0 | -4.299716 | -1.359200 | -0.919467 |
| 25 | 1 | 0 | -4.607450 | -0.517460 | -1.543510 |
| 26 | 1 | 0 | -3.916531 | -2.146358 | -1.572678 |
| 27 | 6 | 0 | -5.429022 | -1.852929 | -0.046372 |
| 28 | 1 | 0 | -5.796566 | -1.055911 | 0.602903 |
| 29 | 1 | 0 | -6.253272 | -2.191311 | -0.678371 |
| 30 | 1 | 0 | -5.105168 | -2.690673 | 0.574756 |
| 31 | 1 | 0 | -0.129357 | -1.093822 | 0.745243 |
| 32 | 8 | 0 | 3.466696 | -3.200481 | -0.171532 |
| 33 | 6 | 0 | 3.182122 | -3.638080 | -1.516443 |
| 34 | 1 | 0 | 3.883928 | -4.444672 | -1.704502 |
| 35 | 1 | 0 | 2.156217 | -3.999653 | -1.580517 |
| 36 | 1 | 0 | 3.342693 | -2.818812 | -2.217098 |
| 37 | 6 | 0 | 2.899842 | -1.619863 | 1.567190 |
| 38 | 6 | 0 | 1.911794 | -0.651915 | 1.607011 |
| 39 | 1 | 0 | 1.692962 | 0.035786 | 2.407415 |
| 40 | 1 | 0 | 3.612879 | -1.893366 | 2.326228 |

**TSM**

| Center Number | Atomic Number | Atomic Type | Coordinates (Angstroms) X | Y | Z |
|---|---|---|---|---|---|
| 1 | 6 | 0 | -1.070773 | -0.034621 | 0.300667 |
| 2 | 6 | 0 | 2.779389 | -2.210340 | 0.314212 |
| 3 | 6 | 0 | 1.155821 | -0.726816 | 0.416695 |
| 4 | 6 | 0 | 0.296797 | 0.285136 | -0.328730 |
| 5 | 1 | 0 | 0.209316 | -0.118394 | -1.341400 |
| 6 | 8 | 0 | 1.809349 | -1.677769 | -0.399209 |
| 7 | 7 | 0 | -1.364379 | 0.612496 | 1.529778 |
| 8 | 8 | 0 | -2.511673 | 0.683223 | 1.959628 |
| 9 | 8 | 0 | -0.399646 | 1.060009 | 2.163694 |
| 10 | 6 | 0 | 0.748052 | 1.722715 | -0.433202 |
| 11 | 6 | 0 | 2.085428 | 2.079845 | -0.285185 |
| 12 | 6 | 0 | -0.183400 | 2.710806 | -0.750896 |
| 13 | 6 | 0 | 2.485873 | 3.402660 | -0.444253 |
| 14 | 1 | 0 | 2.826491 | 1.326332 | -0.040024 |
| 15 | 6 | 0 | 0.213846 | 4.032819 | -0.906738 |
| 16 | 1 | 0 | -1.228736 | 2.447125 | -0.875723 |
| 17 | 6 | 0 | 1.551321 | 4.384005 | -0.752882 |
| 18 | 1 | 0 | 3.530843 | 3.665022 | -0.322478 |
| 19 | 1 | 0 | -0.523694 | 4.790087 | -1.147648 |
| 20 | 1 | 0 | 1.861840 | 5.415854 | -0.871164 |
| 21 | 6 | 0 | -2.153800 | -0.429903 | -0.627738 |
| 22 | 8 | 0 | -2.008263 | -0.386587 | -1.835023 |
| 23 | 8 | 0 | -3.238953 | -0.924551 | -0.046187 |
| 24 | 6 | 0 | -4.299672 | -1.359401 | -0.919468 |
| 25 | 1 | 0 | -4.607432 | -0.517612 | -1.543435 |
| 26 | 1 | 0 | -3.916499 | -2.146501 | -1.572756 |
| 27 | 6 | 0 | -5.428950 | -1.853214 | -0.046382 |
| 28 | 1 | 0 | -5.796445 | -1.056267 | 0.603006 |
| 29 | 1 | 0 | -6.253236 | -2.191501 | -0.678384 |
| 30 | 1 | 0 | -5.105084 | -2.691040 | 0.574629 |
| 31 | 1 | 0 | -0.129218 | -1.093913 | 0.745265 |
| 32 | 8 | 0 | 3.467165 | -3.200041 | -0.171712 |
| 33 | 6 | 0 | 3.182507 | -3.637759 | -1.516566 |
| 34 | 1 | 0 | 3.884368 | -4.444301 | -1.704632 |
| 35 | 1 | 0 | 2.156629 | -3.999429 | -1.580526 |
| 36 | 1 | 0 | 3.342940 | -2.818525 | -2.217291 |
| 37 | 6 | 0 | 2.900226 | -1.619504 | 1.567045 |
| 38 | 6 | 0 | 1.912030 | -0.651720 | 1.606957 |
| 39 | 1 | 0 | 1.693173 | 0.035944 | 2.407384 |
| 40 | 1 | 0 | 3.613395 | -1.892878 | 2.326006 |

**TSN**

| Center Number | Atomic Number | Atomic Type | Coordinates (Angstroms) X | Y | Z |
|---|---|---|---|---|---|
| 1 | 6 | 0 | -0.887896 | -0.978210 | -0.836802 |
| 2 | 6 | 0 | -2.690515 | -2.134783 | -0.346284 |
| 3 | 6 | 0 | -0.074839 | 0.307651 | -0.747771 |
| 4 | 1 | 0 | 0.341857 | 0.414556 | -1.753876 |
| 5 | 6 | 0 | 1.130843 | -0.193110 | 0.067935 |
| 6 | 8 | 0 | -1.867163 | -1.237732 | 0.145114 |
| 7 | 6 | 0 | 2.450175 | -0.076746 | -0.590104 |
| 8 | 8 | 0 | 2.552709 | 0.371076 | -1.719103 |
| 9 | 8 | 0 | 3.484777 | -0.559312 | 0.085634 |
| 10 | 7 | 0 | 0.997517 | -0.191371 | 1.486761 |
| 11 | 8 | 0 | 1.947818 | -0.470411 | 2.212941 |
| 12 | 8 | 0 | -0.125196 | 0.043292 | 1.940935 |
| 13 | 6 | 0 | -0.763335 | 1.604486 | -0.405165 |
| 14 | 6 | 0 | -2.109830 | 1.798120 | -0.704646 |
| 15 | 6 | 0 | -0.033492 | 2.659986 | 0.138521 |
| 16 | 6 | 0 | -2.718773 | 3.024540 | -0.459658 |
| 17 | 1 | 0 | -2.693860 | 0.991092 | -1.134489 |
| 18 | 6 | 0 | -0.639647 | 3.885587 | 0.383285 |
| 19 | 1 | 0 | 1.016800 | 2.524560 | 0.376252 |
| 20 | 6 | 0 | -1.986277 | 4.071959 | 0.086567 |
| 21 | 1 | 0 | -3.768479 | 3.159179 | -0.695684 |
| 22 | 1 | 0 | -0.059176 | 4.695913 | 0.809787 |
| 23 | 1 | 0 | -2.460939 | 5.026912 | 0.281293 |
| 24 | 6 | 0 | 4.766675 | -0.496450 | -0.569588 |
| 25 | 1 | 0 | 4.711612 | -1.057737 | -1.505011 |
| 26 | 1 | 0 | 4.988391 | 0.545506 | -0.809911 |
| 27 | 6 | 0 | 5.785523 | -1.082689 | 0.379142 |
| 28 | 1 | 0 | 5.549852 | -2.123318 | 0.611365 |
| 29 | 1 | 0 | 6.773868 | -1.048430 | -0.084957 |
| 30 | 1 | 0 | 5.821931 | -0.515123 | 1.311149 |
| 31 | 1 | 0 | 0.327753 | -1.384427 | -0.341013 |
| 32 | 8 | 0 | -3.634020 | -2.618124 | 0.404236 |
| 33 | 6 | 0 | -3.771901 | -2.096422 | 1.743730 |
| 34 | 1 | 0 | -4.624095 | -2.621742 | 2.163551 |
| 35 | 1 | 0 | -2.869468 | -2.304565 | 2.317246 |
| 36 | 1 | 0 | -3.959485 | -1.023461 | 1.707539 |
| 37 | 6 | 0 | -2.385811 | -2.474199 | -1.663066 |
| 38 | 6 | 0 | -1.284829 | -1.702485 | -1.981048 |
| 39 | 1 | 0 | -0.758412 | -1.675032 | -2.924067 |
| 40 | 1 | 0 | -2.908608 | -3.198010 | -2.264832 |

**TSO**

| Center Number | Atomic Number | Atomic Type | Coordinates (Angstroms) X | Y | Z |
|---|---|---|---|---|---|
| 1 | 6 | 0 | -0.888036 | -0.978114 | -0.836753 |
| 2 | 6 | 0 | -2.690759 | -2.134594 | -0.346381 |
| 3 | 6 | 0 | -0.074872 | 0.307646 | -0.747606 |
| 4 | 1 | 0 | 0.341831 | 0.414554 | -1.753712 |
| 5 | 6 | 0 | 1.130809 | -0.193306 | 0.067962 |
| 6 | 8 | 0 | -1.867328 | -1.237672 | 0.145130 |
| 7 | 6 | 0 | 2.450091 | -0.076919 | -0.590159 |
| 8 | 8 | 0 | 2.552550 | 0.370856 | -1.719185 |
| 9 | 8 | 0 | 3.484741 | -0.559384 | 0.085562 |
| 10 | 7 | 0 | 0.997603 | -0.191750 | 1.486802 |
| 11 | 8 | 0 | 1.947852 | -0.471320 | 2.212848 |
| 12 | 8 | 0 | -0.124993 | 0.043232 | 1.941102 |
| 13 | 6 | 0 | -0.763205 | 1.604566 | -0.404998 |
| 14 | 6 | 0 | -2.109664 | 1.798381 | -0.704524 |
| 15 | 6 | 0 | -0.033235 | 2.659972 | 0.138699 |
| 16 | 6 | 0 | -2.718441 | 3.024893 | -0.459584 |
| 17 | 1 | 0 | -2.693795 | 0.991430 | -1.134373 |
| 18 | 6 | 0 | -0.639223 | 3.885665 | 0.383412 |
| 19 | 1 | 0 | 1.017026 | 2.524400 | 0.376482 |
| 20 | 6 | 0 | -1.985816 | 4.072222 | 0.086640 |
| 21 | 1 | 0 | -3.768121 | 3.159675 | -0.695649 |
| 22 | 1 | 0 | -0.058654 | 4.695918 | 0.809921 |
| 23 | 1 | 0 | -2.460349 | 5.027247 | 0.281323 |
| 24 | 6 | 0 | 4.766620 | -0.496500 | -0.569696 |
| 25 | 1 | 0 | 4.711550 | -1.057817 | -1.505099 |
| 26 | 1 | 0 | 4.988294 | 0.545457 | -0.810050 |
| 27 | 6 | 0 | 5.785507 | -1.082676 | 0.379034 |
| 28 | 1 | 0 | 5.549908 | -2.123323 | 0.611250 |
| 29 | 1 | 0 | 6.773852 | -1.048344 | -0.085058 |
| 30 | 1 | 0 | 5.821868 | -0.515114 | 1.311046 |
| 31 | 1 | 0 | 0.327628 | -1.384574 | -0.341105 |
| 32 | 8 | 0 | -3.634355 | -2.617890 | 0.404054 |
| 33 | 6 | 0 | -3.772187 | -2.096336 | 1.743608 |
| 34 | 1 | 0 | -4.624510 | -2.621535 | 2.163320 |
| 35 | 1 | 0 | -2.869823 | -2.304748 | 2.317135 |
| 36 | 1 | 0 | -3.959547 | -1.023331 | 1.707553 |
| 37 | 6 | 0 | -2.386047 | -2.473923 | -1.663180 |
| 38 | 6 | 0 | -1.284992 | -1.702274 | -1.981072 |
| 39 | 1 | 0 | -0.758533 | -1.674811 | -2.924067 |
| 40 | 1 | 0 | -2.908891 | -3.197636 | -2.265024 |

**TSP**

| Center Number | Atomic Number | Atomic Type | Coordinates (Angstroms) X | Y | Z |
|---|---|---|---|---|---|
| 1 | 6 | 0 | -1.395070 | -0.566276 | 0.165941 |
| 2 | 6 | 0 | 3.320220 | -1.151926 | -0.145953 |
| 3 | 6 | 0 | 0.937310 | -1.296090 | 0.582864 |
| 4 | 6 | 0 | 0.046112 | -0.317279 | -0.183346 |
| 5 | 1 | 0 | 0.163876 | -0.560687 | -1.239539 |
| 6 | 8 | 0 | 2.266083 | -1.449976 | -0.734006 |
| 7 | 7 | 0 | -1.619377 | -1.109439 | 1.370655 |
| 8 | 8 | 0 | -2.725704 | -1.344344 | 1.873024 |
| 9 | 8 | 0 | -0.553551 | -1.406451 | 2.047915 |
| 10 | 6 | 0 | 0.504620 | 1.119398 | 0.018330 |
| 11 | 6 | 0 | 1.355041 | 1.711339 | -0.911986 |
| 12 | 6 | 0 | 0.125605 | 1.843000 | 1.148042 |
| 13 | 6 | 0 | 1.830258 | 3.003678 | -0.714091 |
| 14 | 1 | 0 | 1.642942 | 1.159778 | -1.801203 |
| 15 | 6 | 0 | 0.599054 | 3.134057 | 1.348220 |
| 16 | 1 | 0 | -0.543551 | 1.396380 | 1.875988 |
| 17 | 6 | 0 | 1.455403 | 3.716819 | 0.418921 |
| 18 | 1 | 0 | 2.487615 | 3.454937 | -1.448837 |
| 19 | 1 | 0 | 0.296779 | 3.687140 | 2.230385 |
| 20 | 1 | 0 | 1.822415 | 4.725050 | 0.574255 |
| 21 | 6 | 0 | -2.405547 | -0.160496 | -0.793427 |
| 22 | 8 | 0 | -2.091159 | 0.341755 | -1.861734 |
| 23 | 8 | 0 | -3.673021 | -0.373405 | -0.441542 |
| 24 | 6 | 0 | -4.676157 | 0.024289 | -1.392960 |
| 25 | 1 | 0 | -4.568208 | 1.092205 | -1.595572 |
| 26 | 1 | 0 | -4.512371 | -0.516721 | -2.327836 |
| 27 | 6 | 0 | -6.023324 | -0.297425 | -0.788813 |
| 28 | 1 | 0 | -6.171101 | 0.246623 | 0.146370 |
| 29 | 1 | 0 | -6.812407 | -0.005942 | -1.485978 |
| 30 | 1 | 0 | -6.116709 | -1.367120 | -0.589971 |
| 31 | 1 | 0 | 0.720964 | -2.352165 | 0.484159 |
| 32 | 8 | 0 | 4.483029 | -1.142388 | -0.714570 |
| 33 | 6 | 0 | 4.543209 | -1.481771 | -2.119554 |
| 34 | 1 | 0 | 5.593348 | -1.406589 | -2.383066 |
| 35 | 1 | 0 | 4.176250 | -2.496318 | -2.267012 |
| 36 | 1 | 0 | 3.946297 | -0.772569 | -2.691665 |
| 37 | 6 | 0 | 3.195386 | -0.798318 | 1.257823 |
| 38 | 6 | 0 | 1.918107 | -0.875698 | 1.623342 |
| 39 | 1 | 0 | 1.551884 | -0.649551 | 2.613498 |
| 40 | 1 | 0 | 4.030828 | -0.513711 | 1.878625 |
